# Disturbed shear stress promotes atherosclerosis through TRIM21‐regulated MAPK6 degradation and consequent endothelial inflammation

**DOI:** 10.1002/ctm2.70168

**Published:** 2025-01-06

**Authors:** Feng Wang, Shu‐Yu Wang, Yue Gu, Shuai Luo, Ai‐Qun Chen, Chao‐Hua Kong, Wen‐Ying Zhou, Li‐Guo Wang, Zhi‐Mei Wang, Guang‐Feng Zuo, Xiao‐Fei Gao, Jun‐Jie Zhang, Shao‐Liang Chen

**Affiliations:** ^1^ Division of Cardiology Nanjing First Hospital, Nanjing Medical University Nanjing China; ^2^ Key Laboratory of Cardiovascular Intervention and Regenerative Medicine of Zhejiang Province, Department of Cardiology, Sir Run Run Shaw Hospital, School of Medicine Zhejiang University Hangzhou China; ^3^ College of Pharmacy Nanjing Medical University Nanjing China

**Keywords:** atherosclerosis, endothelial inflammation, MAPK6, shear stress, TRIM21

## Abstract

**Rationale:**

Coronary artery plaques often develop in regions subjected to disturbed shear stress (DSS), yet the mechanisms underlying this phenomenon remain poorly understood. Our study aimed to elucidate the unknown role of MAPK6 in shear stress and plaque formation.

**Methods:**

In vitro and in vivo experiments, RNA‐seq, CO‐IP and proteomic analysis, combined with single‐cell RNA‐seq datasets were used to reveal the upstream and downstream mechanisms involved. AAV‐MAPK6, ApoE^−/−^MAPK6^flox/flox^TEK^Cre^ mice and the CXCL12 neutraligand were used to confirm the beneficial effects of MAPK6 against atherosclerosis.

**Results:**

Our study revealed a substantial decrease in MAPK6 protein levels in endothelial cells in response to DSS, both in vivo and in vitro, which was contingent on the binding of the ubiquitin ligase TRIM21 to MAPK6. Endothelium‐specific MAPK6 overexpression exerts antiatherosclerotic effects in ApoE^−/−^ mice, elucidating the unexplored role of MAPK6 in atherosclerosis. Comprehensive RNA‐seq, integrated single‐cell mapping and further experiments unveiled the involvement of MAPK6 in inflammation through the EGR1/CXCL12 axis. ApoE^−/−^MAPK6^flox/flox^TEK^Cre^ mice finally confirmed that conditional MAPK6 knockout resulted in endothelial inflammation and significant increases in plaque areas. Notably, these effects could be reversed through the neutralization of CXCL12.

**Conclusions:**

Our study illuminates the advantages of MAPK6 in decelerating plaque progression, highlighting the potential of safeguarding MAPK6 as a novel therapeutic strategy against atherosclerosis.

**Key points:**

- Disturbed flow activates the ubiquitin‒proteasome degradation pathway of MAPK6 in endothelial cells, which is contingent on the binding of the ubiquitin ligase TRIM21 to MAPK6.
- Endothelial MAPK6 has an advantageous impact on decelerating plaque progression.
- MAPK6 regulates endothelial inflammation via the EGR1/CXCL12 axis.

## Graphical abstract

Disturbed flow degrades endothelial MAPK6 through the TRIM21‐regulated ubiquitin‒proteasome pathway, consequently promoting inflammation via EGR1‐mediated nuclear import of NF‐κB p65 and downstream CXCL12 transcription, leading to acceleration of plaque formation.

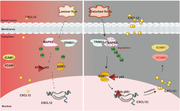

## 1 INTRODUCTION

Despite progress in atherosclerosis diagnosis and treatment, it still remains a significant global health challenge.^1^, ^2^, ^3^ For decades, vascular endothelial cells (ECs) have been recognized to play a key role in atherosclerosis.^4^, ^5^ ECs experiencing wall shear stress from hemodynamic flow respond differently on the basis of flow patterns dictated by vessel geometry. Unidirectional flow with laminar shear stress (LSS) promotes favourable EC morphology with an atheroprotective effect, whereas non‐directional flow with disturbed shear stress (DSS) induces an atherosclerotic EC phenotype characterized by proinflammatory molecule secretion, increased cell turnover, and disrupted cell connections, ultimately contributing to atherosclerosis.^6^, ^7^, ^8^ Thus, a comprehensive understanding of the precise links between mechanical forces and endothelial epigenetic modifications is crucial for pinpointing effective intervention targets in atherosclerosis.

The mitogen‐activated protein kinase (MAPK) signalling pathway is pivotal for cellular responsiveness to external stimuli. In mammals, more than a dozen MAPK species are categorized into classical and noncanonical types. Classical MAPKs, including ERK1/2 (MAPK3/1), JNK1‐3 (MAPK8‐10), p38 (MAPK11‐14), and ERK5 (MAPK7), have been proven to be activated by shear stress and predominantly participate in atherosclerosis progression.^9^, ^10^, ^11^ Unlike classical MAPKs, their noncanonical counterparts including NLK, ERK3 (MAPK6), ERK4 (MAPK4), and ERK7/8 (MAPK15), lack the three‐tier kinase cascade of MAP3K/MAP2K/MAPK.^12^, ^13^, ^14^ The mechanism underlying the involvement of noncanonical MAPKs in shear stress and atherosclerosis remains unclear.

Among all noncanonical MAPKs, we found that only the protein level of MAPK6 is changed by disturbed shear stress. Specifically, a significant reduction in MAPK6 was observed in endothelial cells following DSS stimulation via activation of the MAPK6 ubiquitin‒proteasome degradation pathway, without the detection of phosphorylated MAPK6. This phenomenon is distinct from the increased phosphorylation of classical MAPKs in the atherosclerosis. Previous studies revealed the involvement of MAPK6 in inflammation in the nervous system and cardiomyocytes,^15^, ^16^ suggesting a potential role in atherosclerosis progression. However, the mechanism has not been explored in detail, and its relevance to endothelial cells has received limited attention. Our RNA‐seq results and further experiments revealed that early growth response 1 (EGR1)/CXCL12 axis is the downstream effector of endothelial MAPK6 in DSS‐induced inflammation and atherosclerosis. This was confirmed by the reversal of plaque progression in ApoE^−/−^MAPK6^flox/flox^TEK^Cre^ mice in which the CXCL12 neutraligand was applied. Moreover, we meta‐analysed publicly available single‐cell datasets to validate the role of endothelial MAPK6 in atherosclerosis and identify the regulatory role of MAPK6 in inflammation. Taken together, the activation of the MAPK6‒TRIM21 ubiquitin‒proteasome complex in ECs following DSS stimulation promotes atherosclerosis by regulating endothelial inflammation through the EGR1/CXCL12 axis. These findings hold promise for the prevention and therapeutic intervention of atherosclerosis.

## 2 METHODS

### 2.1 Human tissue

Coronary blood vessels were obtained from heart transplant recipients (without coronary atherosclerosis) at Nanjing First Hospital. All patients have signed the written informed consent. The study was approved by the Ethics Committee of Nanjing First Hospital and conformed to the principles outlined in the Declaration of Helsinki.

### 2.2 Animals

C57BL6 mice, apolipoprotein E‐deficient (ApoE^−/−^) mice and TEK^Cre^ mice were supplied by Cyagen (Suzhou) Biotechnology Co., Ltd. Mapk6^flox/flox^ mice were obtained from Gempharmatech (Jiangsu) Co., Ltd and crossed with ApoE^−/−^ mice and TEK^Cre^ mice, finally yielding the ApoE^−/−^ MAPK6^flox/flox^TEK^Cre/0^ (marked as ApoE^−/−^MAPK6^flox/flox^TEK^Cre^) mice. Littermate ApoE^−/−^MAPK6^flox/flox^ mice were used as the controls. All the animals were fed under specific pathogen‐free conditions at a constant room temperature with a 12 h light/12 h dark cycle.

All male mice were fed with a western diet (WD, 20% protein, 21% fat, and 50% carbohydrate) at age 8 weeks or continue a normal diet (ND). The mice were euthanized by CO_2_ asphyxiation, after which the necessary tissues were removed for subsequent experiments. The study was approved by the Institutional Animal Care and Use Committee of Nanjing First Hospital and conformed to the Guide of the Care and Use of Laboratory Animals (US National Institutes of Health).

### 2.3 Cell culture

Human umbilical vein endothelial cells (HUVECs) (catalogue #8000) were purchased from Zhong Qiao Xin Zhou Biotechnology Co., Ltd. Endothelial cell medium (Cat #1001, ScienCell) was used for in vitro culture. Human coronary artery endothelial cells (HCAECs) (CP‐H087), purchased from Procell Life Science & Technology Co., Ltd., were cultured with CM‐H087. HEK293 cells obtained from ATCC were cultured in 10% FBS‐supplemented high‐glucose DMEM containing 100 U/mL penicillin and 100 µg/mL streptomycin.

### 2.4 Shear stress study in vitro

A parallel‐plate flow channel (NatureThink) was used to generate LSS (25 dyne/cm^2^) and DSS (±4 dyn/cm^2^, 1 Hz).^17^, ^18^ HUVECs and HCAECs (4 × 10^5^ cells) were seeded onto slides (50 mm × 30 mm) coated with fibronectin. The normal ECM was removed when the cell confluence exceeded 90%, and the cells were cultured in a reduced serum medium (2% FBS‐ECM). After 2 h, the slides were placed in the hydromechanical device, and the cells were treated with the set parameters.

### 2.5 Shear stress study in vivo

Eight‐week‐old male mice were used. A total of two types of shear stress regions were selected for comparison. The first was LSS and DSS in normal mice. A comparison was made between the bifurcated area of the aortic arch and the brachiocephalic trunk. The other type is the mouse carotid artery cast model, which results in DSS in the downstream area of the mould. Briefly, under isoflurane anaesthesia, the left carotid artery of each mouse was placed in a cast mould after disinfection, ventral midline incision in the neck and blunt dissection of the left carotid artery. The right carotid artery of each mouse was used as a control. Subcutaneous injections of meloxicam were used for pain relief. After euthanizing the mice by CO_2_ asphyxiation two days post‐surgery, the necessary tissues for subsequent experiments were collected.

### 2.6 Adeno‐associated virus

The adeno‐associated viruses (AAVs) used in the study were purchased from Shanghai Genechem Co., Ltd. Mouse Mapk6 (NM_015806) endothelium‐specific overexpression AAVs were constructed. The vector sequence used was TIEp‐MCS‐EGFP‐3Flag‐SV40 PolyA, and the cloning site was BamHI/BamHI. Six‐week‐old male mice were injected with a single dose of AAV via the tail vein under isoflurane anaesthesia 2 weeks before moulding.

### 2.7 LIT‐927 administration

The CXCL12 neutraligand, LIT‐927 (MedChemExpress, HY‐112709), was dissolved in 10% w/v methyl‐β‐cyclodextrin (MCD; Sigma, #C0926) and delivered by oral gavage (i.g.) for 8 weeks or 2 days (30 mg/kg/day). The same volume of MCD was administered to the control mice.

### 2.8 Cell transfection

When the cells grow to about 60%–70% confluence, siRNA transfection is performed. The siRNA sequences are shown in Table S3. When the cells grow to about 90%–100% confluence, plasmid transfection is performed. The Lipofectamine 3000 Transfection Reagent (Thermo Fisher Scientific) was used for transfection. Thirty‐six hours after siRNA transfection, the cells were subjected to shear stress or directly extracted. Forty‐eight hours after plasmid transfection, the cells were subjected to shear stress or directly extraction.

### 2.9 Western blotting

The following steps were used to conduct the test: (1) extraction of total cell protein (Lysis Buffer for WB/IP Assays, Yeasen) or nuclear and cytoplasmic protein (Nuclear and Cytoplasmic Protein Extraction Kit, Beyotime); (2) protein concentration determination via the BCA method (Pierce BCA Protein Assay Kits, ThermoFisher); (3) gel preparation and electrophoresis via 10% or 12% SDS‐polyacrylamide gel electrophoresis; (4) membrane transferred to polyvinylidene fluoride membranes and blocked with 5% nonfat milk at room temperature for 2 h; (5) primary antibody incubation at 4°C overnight and secondary antibody incubation at room temperature for 2 h after washing three times in TBST; (6) development exposure via ChemiScope S7 (Clinx) and Genegenome XRQ (Genegnome). The antibodies used for western blotting are shown in Table S4.

### 2.10 RNA extraction and qRT‐PCR analysis

Total RNA was extracted via the TRIzol method (Beyotime). The RNA concentration was determined with a NanoDrop 2000. The reagents for RNA reverse transcription and qPCR were purchased from Nanjing Vazyme Biotech Co., Ltd. and were used according to the instructions for reagent use. The primers used for qPCR are shown in Table S5.

### 2.11 H&E staining and Aorta Oil Red staining

H&E staining: The paraffin sections were dewaxed and dehydrated, stained with haematoxylin and eosin, and finally sealed and fixed with neutral glue after dehydration. During progression, if the staining depth was too deep, the differentiation step could be repeated; if the staining depth was too shallow, the staining could be repeated.

Aorta Oil Red O staining: After rinsing with .9% normal saline, the samples were immersed in the prepared Oil Red O solution and shaken at a low speed on a shaking table. When a purplish‐red colour appeared in the plaque, the mixture was incubated with 60% isopropyl alcohol for decolourisation, and the addition of isopropyl alcohol was stopped after no red colour was observed.

### 2.12 Immunofluorescence staining

The slides and cells were fixed with 4% paraformaldehyde and permeabilized with 0.1% Triton X‐100. The samples were washed with PBS three times for 5 min each, after which the samples were sealed with 1% bovine serum albumin at room temperature for 1 h. After incubation with primary antibody overnight at 4°C, the samples were washed with PBS three times for 5 min each. The secondary antibody was applied at room temperature for 2 h in the dark. After three washes with PBS, the nuclei were stained with DAPI, and the samples were observed and photographed under a laser confocal microscope (Zeiss LSM 800). The antibodies used for immunofluorescence (IF) are shown in Table S4. NF‐κB activation and nuclear translocation assay kit (Beyotime) were used to assess the activity of NF‐κB p65.

### 2.13 Serum and supernatant analysis

The supernatant samples were collected by centrifuging the cell culture at 1500 rpm for 10 min at 4°C. Serum samples were collected by centrifuging the blood at 3000 rpm for 15 min at 4°C. Total cholesterol assay kit, high‐density lipoprotein cholesterol assay kit, low‐density lipoprotein cholesterol assay kit, and triglyceride assay kit (Jiancheng Bioengineering Institute) were used to detect lipid concentrations in mouse serum. Human CXCL12 ELISA Kit (Abcam), mouse CXCL12 ELISA Kit (Abcam), TGF‐β ELISA Kit (Abcam), TNF‐α ELISA Kit (Abcam), INF‐γ ELISA Kit (Abcam), and IL‐6 ELISA Kit (Abcam) were used according to the instructions.

### 2.14 RNA‐seq

HUVECs were transduced with si‐NC or si‐MAPK6 for 36 h, after which RNA was extracted. Three randomly selected samples were subsequently sent to OE Biotech, Inc. for RNA sequencing analysis. HISAT2 was used to perform sequence alignment between CleanReads and GRCh38.p13. The sequence similarity alignment method was used to identify the expression abundance of each protein‐coding gene in each sample. The HTSeq count software was used to obtain the number of reads aligned to protein‐coding genes in each sample. After comparing and obtaining counts, the protein‐encoding genes were filtered to remove genes with counts of 0 in all samples. By default, DESeq was used for differential analysis of nonbiological duplicate samples. The thresholds *q*‐value <.05 and |log2FC| > 1 were used to identify DEGs for enrichment analysis. A threshold *p*‐value < .01 and log2FC > 1 were used to identify upregulated genes. The GSE92506, GSE83476, GSE266437, and GSE197366 datasets were downloaded from the GEO database.^19^, ^20^, ^21^ A threshold *p*‐value < .01 and log2FC > 1 were used for identifying upregulated genes via GEO2R. The basic information and links of the public RNA‐seq data are shown in Table S2.

### 2.15 ChIP‐PCR

A ChIP assay kit (Beyotime) was used. The antibodies used are shown in Table S4. The sequences of the primers used for PCR analysis in the ChIP assays are shown in Table S5.

### 2.16 COIP

(1) Magnetic bead pretreatment: The immunomagnetic beads were resuspended with an anti‐label. Magnetic beads were washed with washing liquid. (2) Dressing: Two‐hundred microlitres of cell lysate was added to the precipitate, and the samples were incubated at room temperature for 2 h or 4°C overnight. (3) Remove impurities: After the samples were allowed to stand on a magnetic rack for 10 s, the supernatant was removed, and the precipitate was retained. Magnetic beads were washed with washing liquid. (4) Denaturation: Twenty microlitres of 1×SDS were added to the precipitate obtained above, which was boiled for 10 min, and cooled to room temperature. (5) The supernatant was collected for SDS‒PAGE detection. The extracted protein samples were subsequently sent to Luming Biotech, Inc. for proteomic analysis.

### 2.17 LC‒MS/MS

After enzymolysis and peptide desalination, protein qualitative analysis was performed via Easy nLC 1200 ultra‐high performance liquid chromatography tandem Q Exactive plus high‐resolution mass spectrometer, combined with ProteomeDiscover2.3 data processing software.

### 2.18 Wire myograph

Eight‐week‐old male mice were euthanized by CO_2_ asphyxiation, and the necessary tissues were removed for subsequent experiments. The thoracic aortas of the mice were immersed in cold Krebs‒Henseleit solution, and the surrounding connective tissue was stripped away. The vessels were divided into several 2 mm segments. Wire myograph (Danish Myo Technology, A/S Inc.) was used to measure the tension changes of aortic rings. The tissue segments in the wire myograph were mounted, and the device was switched on to achieve set adjustment (37°C, 95% O_2_ and 5% CO_2_). The aortic rings were stretched to a physiologically relevant preload and left to equilibrate for approximately 40 min. After the viability of the tissue was confirmed, drugs (acetylcholine, 10^−9^–10^−5^ M) were added to the bath. Finally, the data were recorded.

### 2.19 Single‐cell RNA‐seq analysis

Sixty samples from 14 distinct human or mice scRNA‐seq datasets were collected. The basic information and links of the public scRNA‐seq are shown in Table S2. Each library was loaded into R v4.3.0 by Seurat v4.1.0 for quality control, such as filtering low‐quality cells as recommended by the original research^22^, ^23^, ^24^, ^25^, ^26^, ^27^, ^28^, ^29^, ^30^, ^31^ (shown in Table S2), deleting doublets via DoubletFinder v2.0.3 and removing contamination via SoupX v1.6.2. All the data were integrated with Seurat v5.0.1 by applying HarmonyIntegration after dimensionality reduction of the normalized count matrix via PCA. Clustering results obtained via unsupervised graph‐based clustering partitioning were visualized by uniform manifold approximation and projection (UMAP) embedding.

The MAPK6 pathway score was calculated with AddModuleScore and AUCell v1.20.1 and irGSEA v2.1.5 Using Scissor v2.0.0, we identified cell subpopulations that were strongly correlated with the si‐MAPK6 phenotype from the scRNA‐seq data; these cells were named Scissor+ according to the RNA‐seq data of HUVECs transfected with MAPK6 siRNA or not. For homologous conversion, homologene v1.4.68 were applied. Muscat v1.13.1, CellChat v1.6.1, slingshot v2.6.0, and CytoTRACE v0.3.3 were used for in‐depth analysis respectively. SCP v0.4.8 and ggplot2 v3.4.4 were applied for visualization.

### 2.20 Statistics

The normality of continuous data distribution was evaluated via the Kolmogorov‒Smirnov test or the Shapiro‒Wilk test. All continuous variables are presented as the mean ± SEM. Comparisons between two groups or more than two groups were performed via *t*‐tests or ordinary one‐way ANOVA with Dunnett (or Tukey) multiple comparison test, respectively. All the statistical tests were two‐tailed, and *p* < .05 was used to indicate statistical significance.

## 3 RESULTS

### 3.1 The protein level of endothelial MAPK6 decreases in atherosclerosis

**FIGURE 1:**
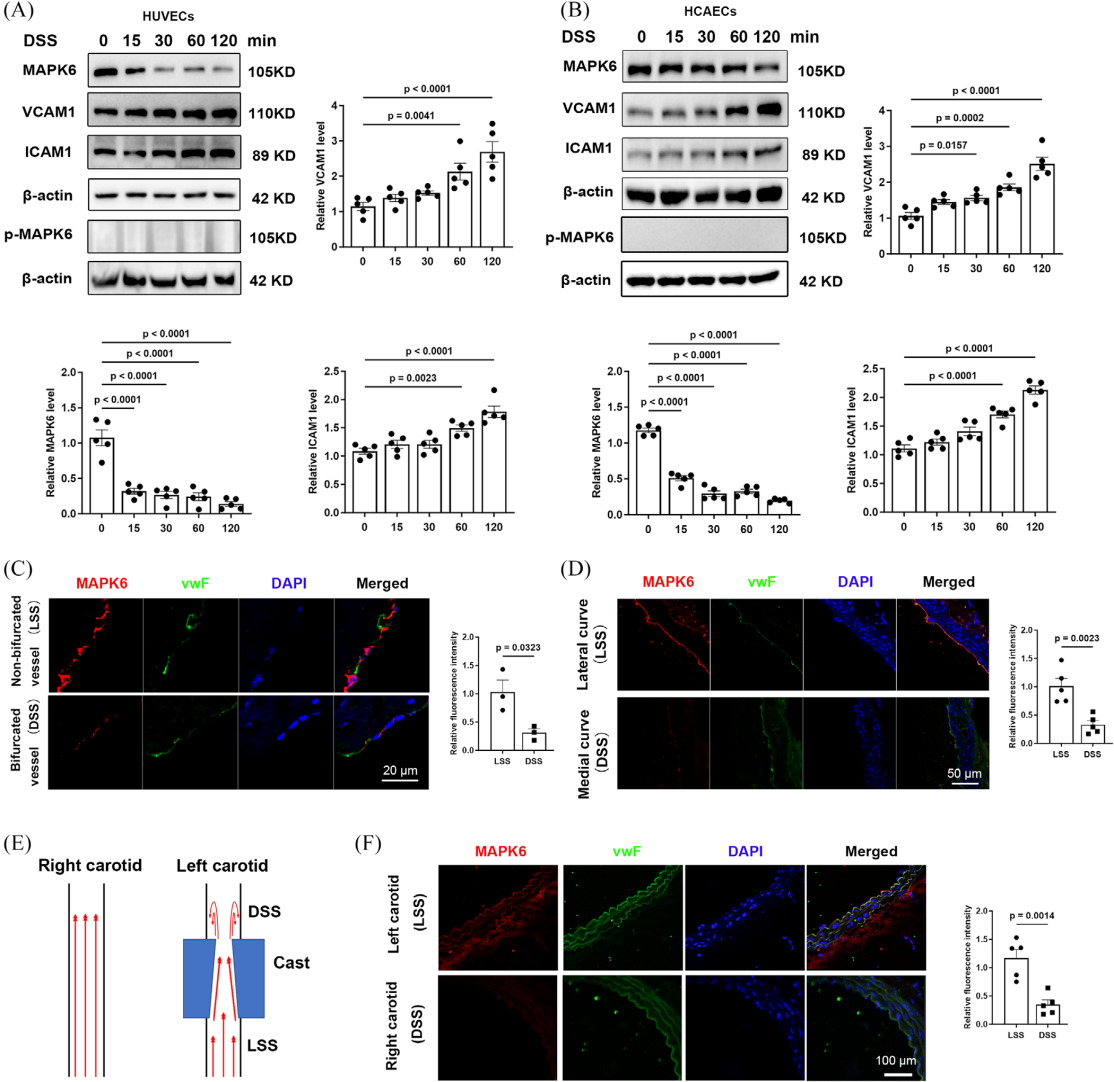
The protein level of endothelial MAPK6 decreases in atherosclerosis. (A) Protein levels of MAPK6, p‐MAPK6, ICAM1, and VCAM1 in HUVECs stimulated with DSS for different durations were detected via WB (*n* = 5). (B) Protein levels of MAPK6, p‐MAPK6, ICAM1, and VCAM1 in HCAECs stimulated with DSS for different durations (*n* = 5). (C) IF staining of non‐bifurcated and bifurcated human coronary vessels (*n* = 3). Red: MAPK6; Green: vWF; Blue: DAPI. Scale bar: 20 µm. (D) IF staining of the lateral curve and medial curve of the aortic arch (*n* = 5). Red: MAPK6; Green: vWF; Blue: DAPI. Scale bar: 50 µm. (E) Diagram of a mouse DSS model. (F) IF staining of the LSS region and DSS region of carotid artery ligation vessels in mice (*n* = 5). Red: MAPK6; Green: vWF; Blue: DAPI. Scale bar: 100 µm. One‐way ANOVA with the Dunnett multiple comparison test was applied to (A) and (B). A two‐tailed Student's *t*‐test was applied for (C), (D), and (F).

After the discovery that DSS stimulation induced a rapid and significant reduction in MAPK6 protein levels in HUVECs before increasing the levels of the inflammatory markers ICAM1 and VCAM1 (Figure 1A), we verified these results in HCAECs and observed the same phenomenon (Figure 1B), whereas the levels of the other MAPK family members did not change (Figure S1 and Table S1). In contrast, MAPK6 levels remained stable after LSS stimulation, even after 72 h of exposure (Figure S2). Several previous studies have reported increased phosphorylation of ERK1, ERK2, and ERK5 in response to shear stress and play a role.^8^, ^32^ However, we noted that MAPK6 (ERK3) exhibited significant changes in total protein levels, with phosphorylated MAPK6‐S189 remaining low and exhibiting no clear trend (Figure 1A,B).

Subsequently, to explore the status of MAPK6 in vivo, we performed IF staining on human coronary bifurcated (DSS region) and nonbifurcated vessels (LSS region). The fluorescence intensity of MAPK6 in ECs within the DSS region was significantly lower than that in the LSS region (Figure 1C). IF staining was subsequently performed on longitudinal sections of the aortic arch of WT mice (LSS in the lateral curve, and DSS in the medial curve; Figure 1D). In the mouse carotid artery cast model (Figure 1E), transverse sections of the cast vessels were used for IF staining, which revealed that the level of endothelial MAPK6 was lower in the left carotid artery (DSS) than in the right carotid artery (LSS; Figure 1F).

### 3.2 Endothelial MAPK6 has anti‐inflammatory effects

**FIGURE 2:**
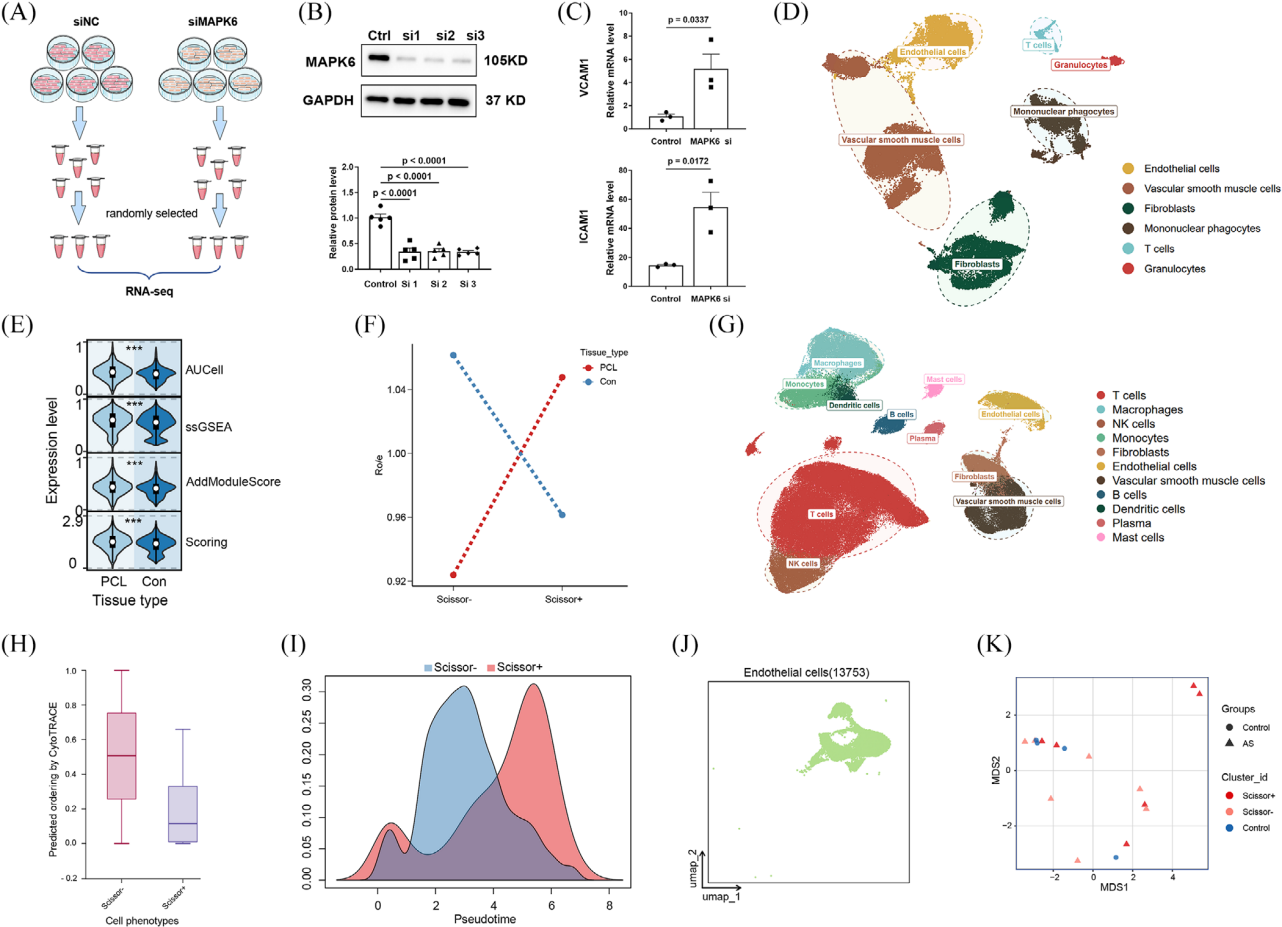
MAPK6 functions through anti‐inflammation. (A) Diagram of the RNA‐seq. (B) Validation of MAPK6 interference efficiency (*n* = 5). (C) The levels of ICAM1 and VCAM1 in the RNA‐seq. (D) UMAP visualization of major cell types in six samples from two mouse PCL single‐cell datasets after pretreatment (see Methods). (E) Pathway score of MAPK6 in ECs according to upregulated genes (*q*‐value < .05 and log2FC > 1) in the si‐MAPK6 group. Scoring = AUCell + AddModuleScore + ssGSEA. (F) Line chart showing the distribution of Scissor+ ECs and Scissor− ECs in samples estimated by the Ro/e score. (G) UMAP embeddings of integrated scRNA‐seq gene expression data of 42 samples from 8 human atherosclerosis single‐cell datasets after pretreatment (see Methods). (H) Analysis of cell differentiation potential of Scissor+ ECs and Scissor− ECs in human plaques by CytoTRACE. (I) Pseudotime analysis of Scissor+ ECs and Scissor− ECs in human plaques by slingshot. (J) UMAP beddings of ECs in normal human aortas. (K) Pseudobulk analysis of Scissor+ ECs, Scissor− ECs and normal ECs applying muscat. A two‐tailed Student's *t*‐test was applied in (C) and (E). One‐way ANOVA with the Dunnett multiple comparison test was applied to (B).

To determine the function of MAPK6 in atherosclerosis, we transfected HUVECs with si‐NC or si‐MAPK6 and extracted RNA for comprehensive RNA sequencing analysis (Figure 2A,B). Differentially expressed genes were identified using a threshold of *q*‐value < .05 and a |log_2_FC| > 1. KEGG, Reactome, and WikiPathways enrichment analyses revealed that MAPK6si upregulated genes related to inflammation (Figure S3A–C). GSEA of several inflammatory pathways also revealed significant inflammatory activation under MAPK6si (Figure S3D). Notably, the expression of the adhesion molecules ICAM1 and VCAM1 substantially increased (Figure 2C).

To preliminarily verify the anti‐inflammation effect of MAPK6 on atherosclerosis in humans, we applied sequencing data from human plaques and ultimately used scRNA‐seq, which can distinguish endothelial cells. Moreover, publicly available scRNA‐seq datasets of mouse carotid arteries in the mouse partial carotid ligation (PCL) model, which is another operative method to induce DSS in vivo were used for the external verification of our conclusions. We retrieved 42 distinct human palgues scRNA‐seq samples and 6 mouse PCL scRNA‐seq sample datasets (Table S2). After quality control, we created meta‐maps via Seurat (details in the Supporting Information Materials). Using UMAP, we identified six major cell types from mouse PCL single‐cell datasets (Figure 2D). The gene expression profiles of the cell types were distinct (Figure S4A). The pathway enrichment score of MAPK6 in ECs according to the upregulated genes in the MAPK6si group revealed enrichment of the ‘MAPK6 pathway’ in ECs under DSS treatment (Figure 2E). By applying the “Scissor” function, we pinpointed EC subpopulations highly correlated with the MAPK6si‐induced phenotype based on our RNA‐seq analysis after homologous conversion. Ro/e analysis further confirmed these findings, showing that Scissor+ ECs were preferentially distributed within regions subjected to DSS, consistent with our experimental results (Figure 2F). Upon conducting an in‐depth analysis of Scissor+ and Scissor− ECs in DSS‐exposed regions of mice, we revealed that Scissor+ ECs interact more actively with other cells in terms of inflammatory pathways (Figure S4B,C).

Similarly, we further analysed 11 cell types of 42 distinct human coronary or carotid plaques scRNA‐seq samples from eight datasets (Figure 2G; Figure S4D). Intriguingly, in plaques, scissor+ ECs interact more actively with other cells in terms of inflammatory pathways (Figure S4E). Similarly, the Scissor+ ECs exhibited an inflammatory activation phenotype (Figure S6F). CytoTRAC and Slingshot analyses were also conducted for pseudotime analysis, revealing a trajectory of ECs from the Scissor− to the Scissor+ group, suggesting a correlation between MAPK6 and disease severity (Figure 2H,I). Considering the lack of available normal human coronary and carotid artery single‐cell RNA sequencing datasets, we collected 12 normal human artery scRNA‐seq samples from 4 datasets and extracted ECs (Figure 4J; Figure S4G,H). Account for the heterogeneity of different blood vessels in humans, we conducted pseudobulk analysis between Scissor+ ECs, Scissor− ECs in human coronary or carotid plaques and normal ECs from normal human aortas, instead of integrating them. MDS analysis revealed that the state of Scissor− ECs was more similar to that of normal ECs, whereas Scissor+ ECs hardly resembled normal ECs (Figure 2K).

### 3.3 Endothelium‐specific MAPK6 overexpression inhibits the progression of atherosclerosis

**FIGURE 3:**
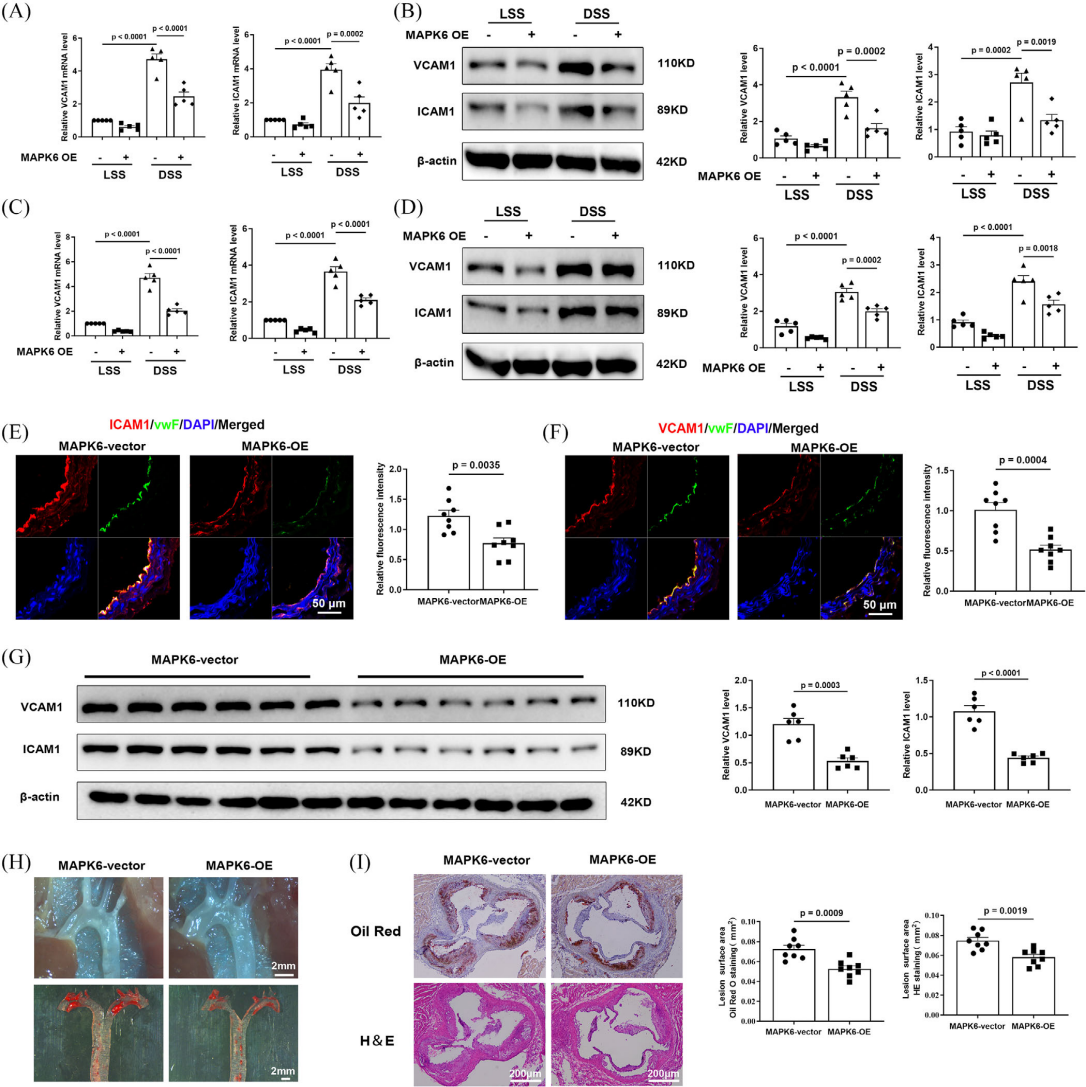
Endothelium‐specific MAPK6 overexpression inhibits the progression of atherosclerosis. (A) The mRNA and (B) protein levels of ICAM1 and VCAM1 in HUVECs after MAPK6 overexpression and 6 h DSS or LSS treatment (*n* = 5). (C) The mRNA and (D) protein levels of ICAM1 and VCAM1 in HCAECs after MAPK6 overexpression and 6 h DSS or LSS treatment (*n* = 5). (E) IF staining of the descending aorta (*n* = 8). Scale bar: 50 µm. Red: ICAM1; Green: vWF; Blue: DAPI. (F) IF staining of the descending aorta (*n* = 8). Scale bar: 50 µm. Red: VCAM1; Green: vWF; Blue: DAPI. (G) Protein levels of ICAM1 and VCAM1 in left carotid artery samples of mouse carotid artery cast model (*n* = 6). (H) Photos of the aortic arch and aortic Oil red O staining of mice after 8 weeks of WD feeding. Scale bar: 2 mm. (I) H&E and Oil red O staining of aortic roots after 8 weeks of WD feeding (*n* = 8). Scale bars: 200 µm. A two‐tailed Student's *t*‐test was applied for (E–H). One‐way ANOVA with the Tukey multiple comparison test was applied for (A–D).

We established, through multiple findings, that MAPK6 expression decreases in response to proatherosclerotic factors and within atherosclerotic tissues. Additionally, MAPK6 has an anti‐inflammation effect. However, the precise relationship between MAPK6 and atherosclerosis has not been determined. In vitro, a human MAPK6‐OE plasmid was constructed and transfected into HUVECs (Figure S5A). The overexpression of MAPK6 in ECs greatly reduced the DSS‐induced increase of ICAM1 and VCAM1 in HUVECs (Figure 3A,B) and HCAECs (Figure 3C,D). In vivo, the endothelium‐specific AAV carrying MAPK6 cDNA for overexpression (referred to as MAPK6‐OE) was generated. A simplified flowchart detailing the model is provided in Figure S5B. Successful endothelium‐specific MAPK6‐OE was confirmed by en face staining (Figure S5C). IF staining of the descending aorta serial section in mice showed that the ECs of ApoE^−/−^ MAPK6‐OE group presented a more severe inflammatory phenotype (Figure 3E,F). In the mouse carotid artery cast model, six left carotid artery samples were randomly selected from each group, and the downregulation of ICAM1 and VCAM1 was found in the ApoE^−/−^ MAPK6‐OE group (Figure 3G). After 8 weeks of WD feeding, the ApoE^−/−^ MAPK6‐OE group exhibited significantly fewer and smaller plaques than did the ApoE^−/−^ group (Figure 3H). We transversely sectioned and stained the aortic sinus region of the mice with haematoxylin and eosin (H&E) and Oil red O (Figure 3I). These results revealed that the average lesion area in the aortic sinus of ApoE^−/−^ MAPK6‐OE mice was significantly smaller than that of ApoE^−/−^ mice. Importantly, MAPK6‐OE did not have any discernible effect on the body weight (Figure S5D). Lipid profiling revealed a significant reduction in triglyceride (TG) levels in the ApoE^−/−^ MAPK6‐OE group, whereas there were no significant differences in LDL‐c, HDL‐c, or total cholesterol (TC) levels (Figure S5E).

### 3.4 DSS promotes the binding of TRIM21 to MAPK6 and degrades MAPK6 through the ubiquitin‒proteasome pathway

**FIGURE 4:**
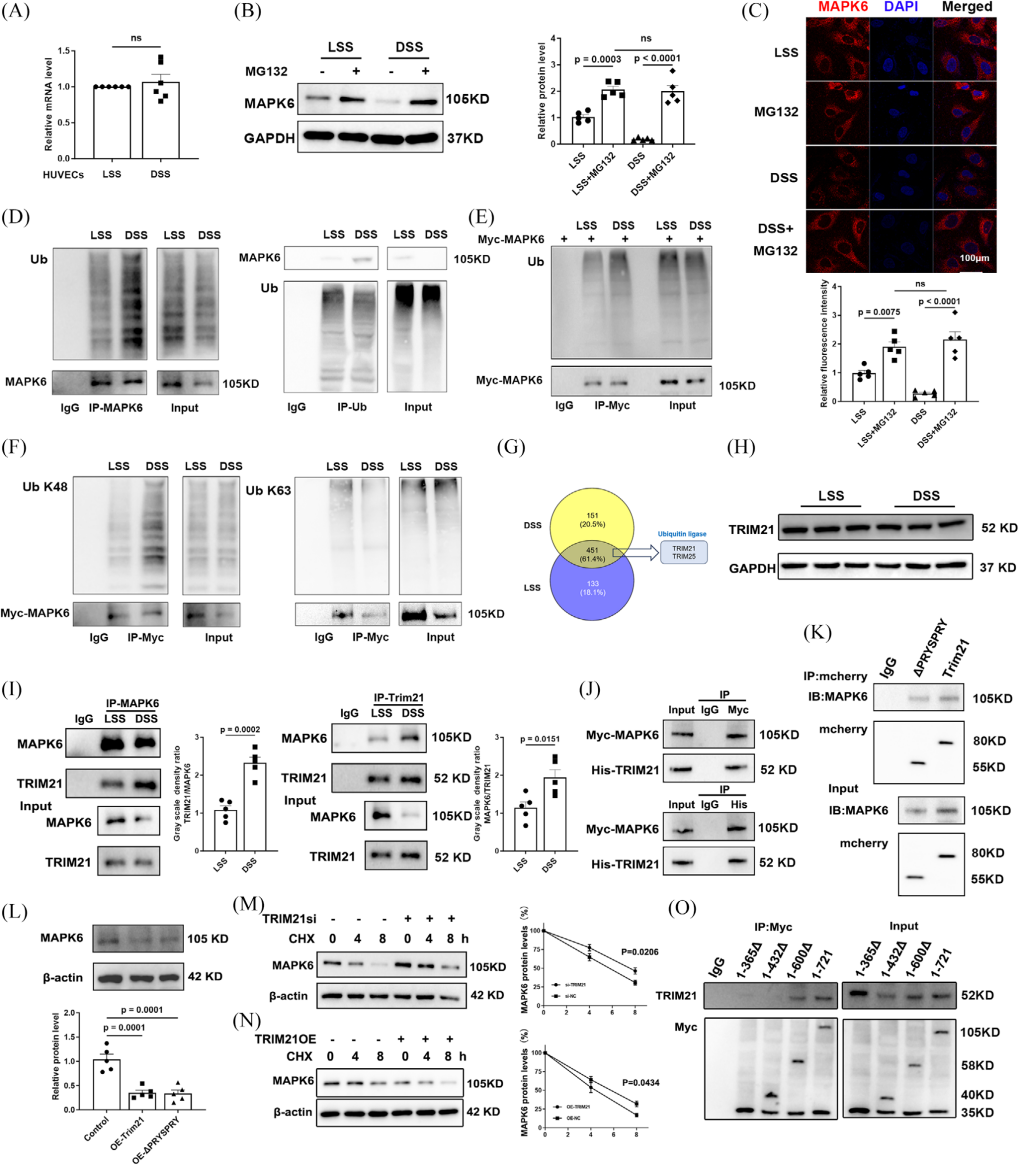
DSS promotes the binding of TRIM21 to MAPK6 and degrades MAPK6 through the ubiquitin‒proteasome pathway. (A) The mRNA level of MAPK6 in HUVECs after DSS and LSS stimulation (*n* = 6). (B) MAPK6 levels of HUVECs stimulated with LSS or DSS (6 h) with or without MG132 (*n* = 5). (C) MAPK6 fluorescence intensity for HUVECs stimulated with LSS or DSS (6 h) with or without MG132 (*n* = 5). Scale bars: 100 µm. (D) WB for MAPK6 and Ub in the input and immunoprecipitates of IgG or anti‐MAPK6 (Ub) antibody in HUVECs treated with LSS or DSS (*n* = 5). (E, F) Myc and Ub (or K48 Ub, K63 Ub) in the input and immunoprecipitates of IgG or anti‐Myc in HUVECs transfected with the Myc‐MAPK6 plasmid (*n* = 5). (G) Venn diagram showing the IP results combined with mass spectrometry screening of MAPK6‐binding proteins. (H) MAPK6 and TRIM21 in the input and immunoprecipitates of the IgG or anti‐MAPK6 (TRIM21) antibody in HUVECs treated with LSS or DSS (*n* = 5). (I) The protein levels of TRIM21 in HUVECs treated with LSS or DSS (*n* = 5). (J) Myc and His in the input and immunoprecipitates of IgG or anti‐Myc in HEK293T cells transfected with the Myc‐MAPK6 and His‐TRIM21 plasmids (*n* = 5). (K) MAPK6 and mCherry in the input and immunoprecipitates in HEK293T cells transfected with different plasmids (*n* = 5). (L) MAPK6 in HUVECs transfected with the pCDNA3.1 mCherry‐TRIM21 and mCherry‐TRIM21ΔPRYSPRY plasmids (*n* = 5). (M, N) MAPK6 in HUVECs transfected with TRIM21si or TRIM21 plasmids and then treated with CHX for 0, 4, or 8 h (*n* = 5). (O) TRIM21 in HUVECs transfected with different plasmids and then enrichment by anti‐Myc antibody (*n* = 5). ns = no significance. A two‐tailed Student's *t*‐test was applied to (A). One‐way ANOVA with the Tukey multiple comparison test was applied to (B) and (C). One‐way ANOVA with the Dunnett multiple comparison test was applied to (L). Two‐way ANOVA with the Sidak multiple comparison test was used for (M) and (N).

The decrease in MAPK6 protein levels may be attributed to two potential factors, a reduction in mRNA synthesis or an increase in protein degradation, specifically involving the ubiquitin‒proteasome degradation pathway and autophagy degradation pathway.^33^, ^34^ To investigate these possibilities, we initially examined MAPK6 mRNA levels, and intriguingly, observed that DSS did not exert any regulatory effect on mRNA expression (Figure 4A). To validate the exact mechanism for the degradation of MAPK6, we applied the inhibitor of ubiquitin‒proteasome degradation pathway, MG132, and the inhibitor of the autophagy degradation pathway, NH_4_Cl. Specifically, levels may be attributed primarily to ubiquitination‐mediated degradation (Figure 4B; Figure S6A). IF analysis of the HUVECs also revealed that the decrease in MAPK6 levels was mitigated by MG132 (Figure 4C). Additionally, immunoprecipitation (IP) experiments targeting endogenous MAPK6 and ubiquitin (Ub) were conducted. After exposure to DSS, we observed an increase in the interaction between MAPK6 and Ub (Figure 4D). Furthermore, we introduced exogenous Myc‐MAPK6 into HUVECs and observed increased binding of exogenous Myc‐MAPK6 to Ub (Figure 4E). In HUVECs transfected with exogenous Myc‐MAPK6, we used IgG and Myc antibodies to precipitate K48Ub and K63Ub, revealing that DSS promoted the binding of MAPK6 to K48Ub (Figure 4F).

The protein binding to MAPK6 was enriched after exposure to LSS or DSS and was used for protein profiling. According to the mass spectrometry data. A total of 451 identical proteins were identified in the two groups, of which two were ubiquitin ligases (Figure 4G). Subsequently, in HUVECs (Figure 4H), we demonstrated that DSS significantly increased the binding of endogenous MAPK6 to TRIM21 (while TRIM25 remained unchanged, as shown in Figure S6B), without changing the protein level of TRIM21 (Figure 4I). In addition, we investigated the interaction between exogenous Myc‐MAPK6 and His‐TRIM21 in HEK 293T cells (Figure 4J). Furthermore, we discovered that both mCherry‐labelled TRIM21 and ΔPRYSPRY TRIM21 bound to MAPK6 (Figure 4K). Upon overexpressing mCherry‐labelled TRIM21 and ΔPRYSPRY TRIM21 in HUVECs, we observed an equivalent reduction in MAPK6 levels (Figure 4L). TRIM21si (the efficacy of TRIM21si is shown in Figure S6C) significantly inhibited MAPK6 degradation in the presence of CHX (Figure 4M). Conversely, the TRIM21‐OE accelerated MAPK6 degradation (Figure 4N). Further investigation involving the construction of MAPK6 subclones with different amino acid fragments tagged with Myc revealed that the crucial binding region with TRIM21 was located within the 432–600 amino acid sequence of MAPK6 (Figure S6D; Figure 4O).

### 3.5 MAPK6 regulates proatherogenic factors through CXCL12

**FIGURE 5:**
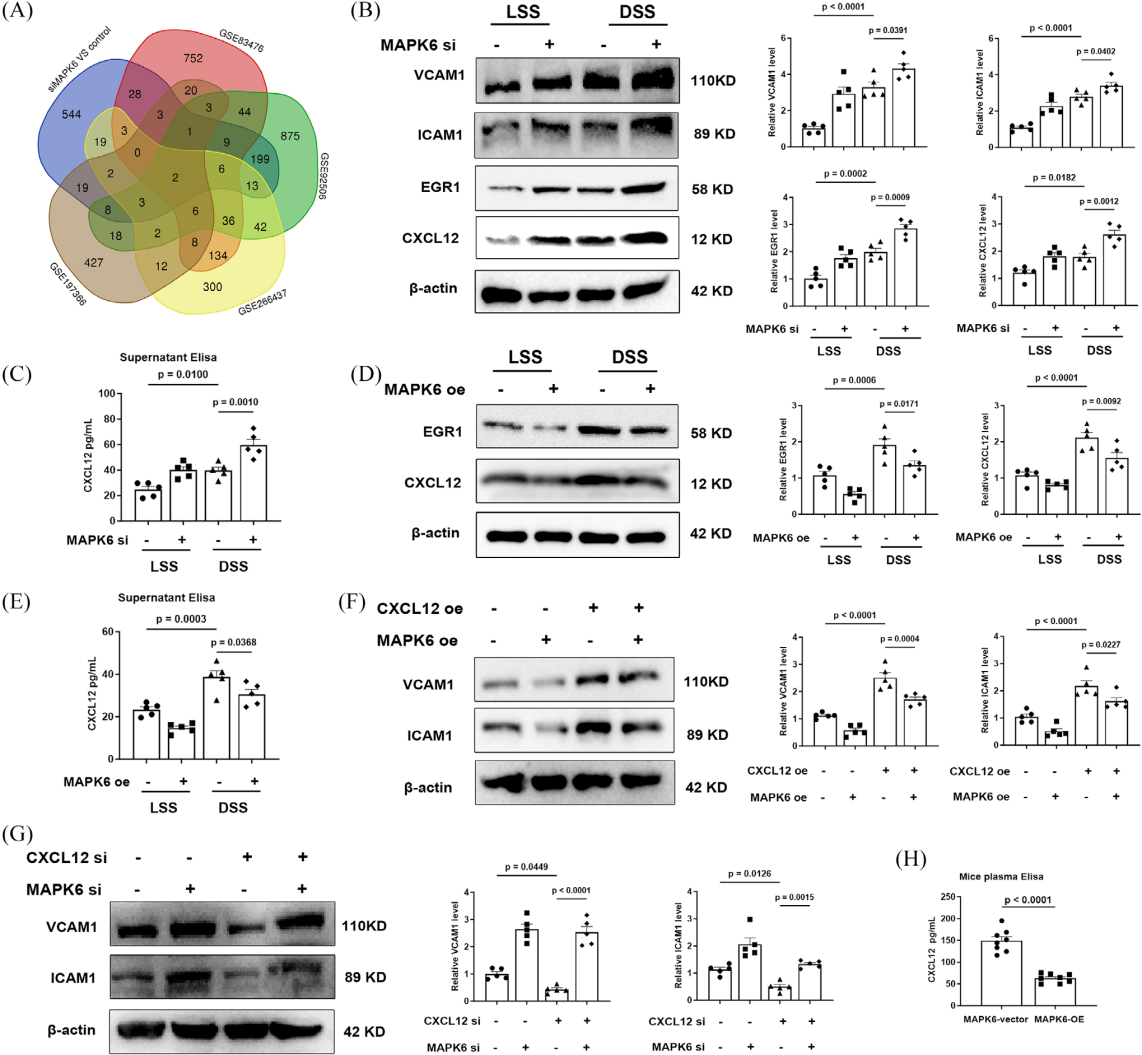
MAPK6 regulates proatherogenic factors through CXCL12. (A) Venn diagram of unregulated genes in si‐NC and si‐MAPK6 groups, GSE92506, GSE83476, GSE197366, and GSE266437. The threshold *p*‐value < .01 and log_2_FC > 1 were used for identifying unregulated genes. The basic information and links of the public RNA‐seq were shown in Table S2. (B) ICAM1, VCAM1, CXCL12, and EGR1 in HCAECs transfected with the MAPK6si or NC and treated with LSS or DSS (6 h) (*n* = 5). (C) CXCL12 in the supernatant of HCAECs transfected with MAPK6si or NC and treated with 6 h LSS or DSS (*n* = 5). (D) Protein levels of CXCL12 and EGR1 in HCAECs transfected with the MAPK6 or NC plasmid and treated with 6 h LSS or DSS (*n* = 5). (E) CXCL12 in the supernatant of HCECs which transfected with the MAPK6 or NC plasmid and treated with 6 h LSS or DSS (*n* = 5). (F) ICAM1 and VCAM1 in HCAECs transfected with the MAPK6 and/or CXCL12 plasmids (*n* = 5). (G) ICAM1 and VCAM1 in HCAECs transfected with the siMAPK6 and/or siCXCL12 (*n* = 5). (H) Plasma CXCL12 levels of mice detected via ELISA kits (*n* = 8). One‐way ANOVA with the Tukey multiple comparison test was applied to (B–G). A two‐tailed Student's *t*‐test was applied in (H).

Considering that DSS induces ECs to transform into proinflammatory phenotypes to accelerate atherosclerosis and that MAPK6si‐upregulated genes are related to inflammation, we focused on the upregulated genes identified in the RNA‐seq. To identify the inflammation‐related genes regulated by MAPK6 in endothelial cells under shear stress, we downloaded four RNA‐seq datasets, namely, GSE92506, GSE83476, GSE266437, and GSE197366. The differentially expressed genes between HUVECs, HCAECs, and HCECs subjected to physiological and pathological shear stress as well as endothelial enriched RNAs of the LCA and RCA in 8‐week male C57BL6 mice subjected to partial carotid ligation surgery were analysed (Figure 5A). The results revealed that CXCL12 and EGR1 were most significantly regulated by MAPK6.

CXCL12 is a secreted protein secreted by the stromal cells including endothelial cells and plays a role in promoting atherosclerosis by intensifying multiple pathogenesis of atherosclerosis, including inflammation, dyslipidemia and so on.^35^ The serum CXCL12 concentration is also closely related to atherosclerosis.^36^, ^37^ Therefore, we tested the protein levels in cells and the concentration of CXCL12 in the cell supernatant and found that DSS promoted the generation of CXCL12 and EGR1, which are reversed by MAPK6 overexpression and exacerbated by MAPK6 downregulation (Figure 5B–E). After verification of CXCL12si and CXCL12 plasmids (Figure S7), we confirmed that the overexpression of CXCL12 in ECs strongly reversed the decrease of ICAM1 and VCAM1 due to MAPK6 overexpression, otherwise the opposite (Figure 5F,G). And we tested the concentration of CXCL12 in the mouse plasma, the results indicated that endothelium‐specific MAPK6 overexpression reduced serum levels of CXCL12 (Figure 5H).

### 3.6 Degradation of MAPK6 promotes endothelial inflammation by activating the EGR1/NF‐κB/CXCL12 pathway

Transcription factor EGR1 also plays a crucial regulatory role in various cardiovascular pathological processes including the pathogenesis of atherosclerotic lesions.^38^ Moreover, CXCL12 expression is largely noncanonical NF‐κB‐dependent in ECs^39^, ^40^, ^41^ which is controlled by protein interactions between NF‐κB and EGR1.^42^ Specifically, EGR1 combines with NF‐κB to accelerate the accumulation of NF‐κB in the nucleus, thus executing the transcription of CXCL12.^42^

**FIGURE 6:**
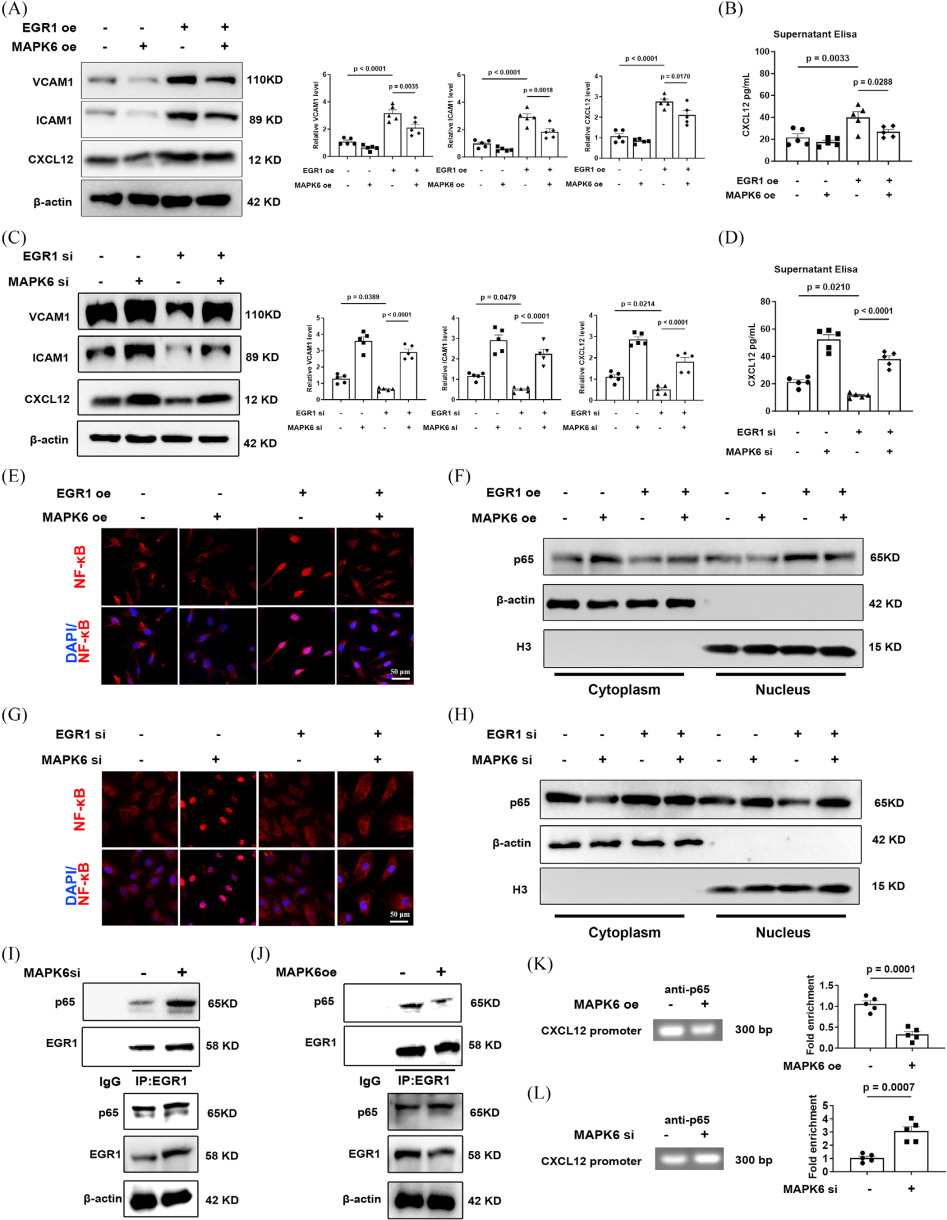
Degradation of MAPK6 promotes endothelial inflammation by activating EGR1/NF‐κB/CXCL12 pathway. (A) CXCL12, ICAM1, and VCAM1 in HCAECs transfected with the MAPK6 and/or EGR1 plasmids (*n* = 5). (B) CXCL12 in the supernatant of HCAECs transfected with the MAPK6 and/or EGR1 plasmids (n = 5). (C) CXCL12, ICAM1 and VCAM1 in HCAECs transfected with the siMAPK6 and/or siEGR1 (*n* = 5). (D) CXCL12 in the supernatant of HCAECs transfected with the siMAPK6 and/or siEGR1 (*n* = 5). (E) Nuclear translocation and activity of NF‐κB p65 in HCAECs transfected with the MAPK6 and/or EGR1 plasmids. Red: NF‐κB p65; Blue: DAPI. Scale bar: 50 µm. (F) Nucleoplasm distribution of NF‐κB p65 in HCAECs transfected with the MAPK6 and/or EGR1 plasmids (*n* = 5). (G) Nuclear translocation and activity of NF‐κB p65 in HCAECs transfected with the siMAPK6 and/or siEGR1. Red: NF‐κB p65; Blue: DAPI. Scale bar: 50 µm. (H) Nucleoplasm distribution of NF‐κB p65 in HCAECs transfected with the siMAPK6 and/or siEGR1 (*n* = 5). (I) NF‐kB in the input and immunoprecipitates of the IgG or anti‐EGR1 antibody in HCAECs transfected with the siMAPK6 or siNC (*n* = 5). (J) NF‐κB in the input and immunoprecipitates of the IgG or anti‐EGR1 antibody in HCAECs transfected with the MAPK6 or NC plasmids (*n* = 5). (K) ChIP assay analysis of the levels of CXCL12 promoter region enriched by NF‐κB in HCAECs transfected with the MAPK6 or NC plasmids (*n* = 5). (L) ChIP assay analysis of the levels of CXCL12 promoter region enriched by NF‐κB in HCAECs transfected with the siMAPK6 or siNC (*n* = 5). A two‐tailed Student's *t*‐test was applied to (K) and (L). One‐way ANOVA with the Tukey multiple comparison test was applied to (A–D).

After verification of EGR1si and EGR1 plasmids (Figure S8), we confirmed that the expression and secretion of endothelial CXCL12 is regulated by the MAPK6/EGR1 axis, and the overexpression of EGR1 reverse the MAPK6‐contributed decrease of ICAM1 and VCAM1, whereas downregulation of EGR1 had an opposite effect (Figure 6A–D). After either decreasing or overexpressing MAPK6, the nuclear accumulation and activity of NF‐κB also increased or decreased respectively, which can be reversed by decreasing or overexpressing EGR1 (Figure 6E–H). Additionally, a decrease/increase in MAPK6 enhances/weakens the interaction between EGR1 and NF‐κB (Figure 6I,J), leading to an increase/decrease in the level of CXCL12 promoter region enriched by NF‐κB (Figure 6K,L). These findings indicated that MAPK6 regulates the interaction between EGR1 and NF‐κB via EGR1, further affecting on the nuclear transport of NF‐κB which plays a critical role in the transcription of CXCL12. In addition, the effect of TRIM21 on the EGR1‐CXCL12 pathway is consistent with the consequence of MAPK6 degradation (Figure S9).

### 3.7 Neutralization of CXCL12 reversed the plaque progression induced by conditional knockout of endothelial MAPK6

**FIGURE 7:**
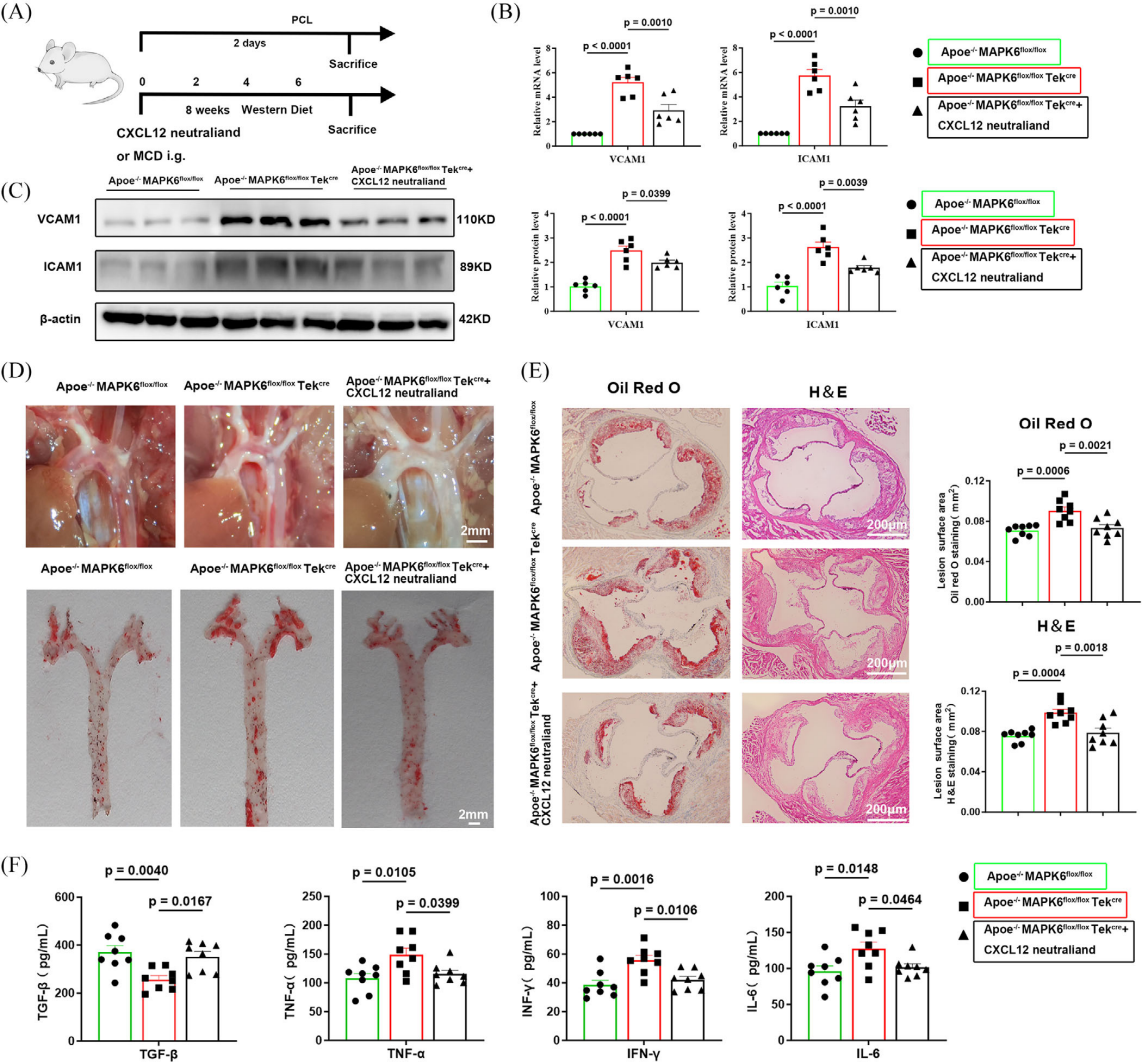
Conditional knockout of endothelial MAPK6 promotes plaque progression which is reversed by neutralization of CXCL12. (A) Schematic diagram of the mouse model. (B) ICAM1 and VCAM1 mRNA levels of left carotid artery samples in the mouse carotid artery cast model (*n* = 6). (C) Protein levels of ICAM1 and VCAM1 in left carotid artery samples in the mouse carotid artery cast model (*n* = 6). (D) Photos and Oil red O staining of the aortic arch after 8 weeks of WD feeding. Scale bar: 2 mm. (E) H&E and Oil red O staining of aortic roots after 8 weeks of WD feeding (*n* = 8). Scale bars: 200 µm. (F) TGF‐β, TNF‐α, IFN‐γ, and IL‐6 levels in mouse plasma after 8 weeks of WD feeding (*n* = 8). One‐way ANOVA with the Tukey multiple comparison test was applied to (B), (C), (E), and (F).

To verify the MAPK6/CXCL12 axis, we generated ApoE and endothelial‐specific MAPK6 dual deficiency mice and applied the CXCL12 neutraligand LIT‐927, which has oral activity and better efficacy than conventional CXCL12‐CXCR4 blockers.^43^, ^44^ A wire myograph experiment demonstrated that MAPK6 endothelial KO did not affect vasoactivity (endothelial‐dependent relaxation, Figure 7A). The subsequent experimental design flowchart is presented in Figure 7B. In the mouse carotid artery cast model, six left carotid artery samples were randomly selected from each group, and the upregulation of ICAM1 and VCAM1 was partially reversed following the LIT‐927 i.g. at both mRNA and protein levels (Figure 7C,D). Compared with those in the ApoE^−/−^ MAPK6^flox/flox^ Tek^Cre^ group, the number of visible plaques in the ApoE^−/−^ MAPK6^flox/flox^ group was significantly greater. However, LIT‐927 reversed plaque progression induced by MAPK6 cKO. Aortic Oil red O staining also corroborated these findings (Figure 7E). Moreover, the aortic sinus region of the mice was dissected and stained with H&E and Oil Red O, and the trend was consistent (Figure 7F). TGF‐β, TNF‐α, IFN‐γ, and IL‐6 levels in mouse plasma after 8 weeks of WD feeding were tested, further confirming that MAPK6 regulated CXCL12 generation to promote endothelial inflammation (Figure 7G). Interestingly, ApoE^−/−^ MAPK6^flox/flox^ Tek^Cre^ mice only presented significantly increased TG levels, whereas after neutralization of CXCL12, all blood lipid levels remained unchanged (Figure S10A). The unchanged weight data of the mice are presented in Figure S10B.

## 4 DISCUSSION

Clinical experience indicates that atherosclerotic plaques tend to occur in arterial bend areas, where blood flow manifests as low shear stress with an irregular direction. An increasing number of studies have demonstrated the initiating role of disturbed flow in atherosclerosis. ECs, residing within the bloodstream, endure hemodynamic forces, including the frictional consequences of fluid shear stress. Accordingly, understanding flow mechanotransduction in ECs subjected to shear stress is critical for revealing the complexities of atherosclerosis.^4^, ^45^ Although the shear stress in ECs has been studied over the past few decades, we still do not understand the mechanisms underlying flow mechanotransduction in ECs well enough to able to treat or prevent the atherosclerosis.

Our study successfully identified MAPK6 as a key factor regulated by mechanical shear stress that significantly influences the inflammatory response of ECs. These insights substantially contribute to understanding the underlying mechanisms triggering atherosclerotic lesions in response to blood flow, offering promising avenues for targeted interventions focused on MAPK6 to prevent and treat atherosclerosis.

Previous studies on MAPK6 have focused predominantly on its implications in oncology,^46^, ^47^, ^48^ where both in vitro and in vivo studies have consistently underscored its role in enhancing the migration and invasion capabilities of cancer cells. In contrast to its well‐established role in tumorigenesis, our study reveals a distinctive and predominantly protective function of MAPK6 in the atherosclerotic process. Specifically, under the influence of DSS, MAPK6 levels in ECs significantly decrease, accompanied by the absence of MAPK6 phosphorylation. Importantly, in vivo experiments confirmed the consequential delay in plaque progression following the endothelium‐specific MAPK6‐OE. This novel finding strongly correlates kinase‐independent signalling mechanisms of MAPK6 with the dynamics of atherosclerotic disease. The stark dichotomy observed in the role of MAPK6 between cancer and atherosclerosis highlights the nuanced and context‐dependent nature of its functions in distinct physiological processes.

MAPK6 lacks a conserved Gly‐Pro‐Glu motif in its C‐terminus, where the key Glu is required for the maintenance of space‐stable folding of the kinase.^49^ We hypothesized that the rapid decrease in MAPK6 in ECs after DSS stimulation is related to the C‐terminus of MAPK6. To investigate the degradation mechanism, we observed that MG132 inhibited MAPK6 degradation via the ubiquitin‒proteasome pathway induced by DSS. Through validating the related ubiquitin ligase TRIM21 through protein profiling and further experiments, our findings add a layer of understanding to the ubiquitination‐mediated degradation of MAPK6. Interestingly, in addition to TRIM21, FBXW7 has been identified as another E3 ligase responsible for the ubiquitination of MAPK6.^50^ This conclusion was drawn from mammalian two‐hybrid experiments and IP results. Our specific findings indicate that MAPK6 interacts with the ubiquitin ligase TRIM21,^51^ forming a complex via the 432–600 region of its amino acid sequence. This interaction mediates the swift ubiquitination‐mediated degradation of MAPK6, shedding light on a critical aspect of the regulatory mechanism governing MAPK6 levels in ECs exposed to DSS.

Previous studies have highlighted the activation of classical MAPKs and NF‐κB in response to LPS stimulation, with the absence of MAPK6 inhibiting the production and secretion of IL‐8 in various cell types.^52^ Consistent with these findings, our experimental data revealed a significant increase in the levels of proinflammatory factors and a decrease in anti‐inflammatory molecules following MAPK inhibition. Our RNA‐seq analysis and further experiments uncovered a direct correlation between diminished MAPK6 expression and a consequential increase in the generation and secretion of CXCL12. CXCL12, also known as SDF‐1, has been proven to be the driving factor of atherosclerosis. Although CXCL12 from other cells, such as adipocytes, immune cells or the liver, may produce important components with unknown functional relevance, CXCL12 derived from arterial endothelial cells is the key contributor to plaques.^35^ The execution of CXCL12 transcription by the typical transcription factor NF‐κB is dependent on the nuclear accumulation of NF‐κB, which can be controlled by protein interactions between NF‐κB and EGR1.^40^, ^41^, ^42^ Our results indicated that DSS stimulation degrades MAPK6 and promotes the interaction between EGR1 and NF‐κB, ultimately accelerating the accumulation of NF‐kB nuclei, thus executing the transcription of CXCL12 via NF‐κB.

Lipid accumulation and inflammation are two pivotal pathogenic factors in atherosclerosis.^43^, ^53^, ^54^ Among the laboratory‐generated mice, although a regulatory effect of MAPK6 on cholesterol levels (except TG) was observed, the statistical significance was limited by the sample size. Interestingly, MAPK6 conditional knockout mice/endothelium‐specific overexpression mice presented significantly increased/decreased TG levels, indicating that MAPK6 may play a unique role in the regulation of TGs. However, the neutralization of CXCL12 cannot reverse the increase in TG caused by conditional knockout of MAPK6 in the endothelium. In macrophages, CXCL12 has been proven to inhibit ABCA1‐dependent cholesterol efflux and aggravate atherosclerosis.^55^, ^56^ On the basis of the results of our experiments that the MAPK6/CXCL12 axis in the endothelium may have little effect on blood lipids and contribute to atherosclerosis largely by promoting inflammation, whereas MAPK6 in the endothelium may affect triglyceride metabolism through other pathways, which may require further exploration in the future. For example, MAPK6 in the liver microvascular endothelial cells may regulate triglyceride metabolism.

In summary, the activation of the MAPK6‒TRIM21 ubiquitin‒proteasome complex in ECs following DSS stimulation highlights the intricate mechanisms involving MAPK6. MAPK6 regulates inflammatory factors in ECs and modulates the expression of adhesion molecules such as ICAM1 and VCAM1 through the CXCL12. In detail, the decrease in MAPK6 expression promotes the interaction between EGR1 and NF‐κB and ultimately accelerates the accumulation of NF‐κB in the nucleus, which promotes the transcription of CXCL12, thereby accelerating the progression of atherosclerosis. These findings hold promise for the prevention and therapeutic intervention of atherosclerosis. The antagonistic effect of MAPK6 on inflammation underscore its potential as a therapeutic target for atherosclerosis, warranting further verification in the context of atherosclerosis.

## AUTHOR CONTRIBUTIONS

Feng Wang, Shu‐Yu Wang, and Yue Gu carried out the major experimental work. Shuai Luo, CAY, Chao‐Hua Kong, Wen‐Ying Zhou, Li‐Guo Wang, Zhi‐Mei Wang, and Guang‐Feng Zuo participated in the experiment at different stages. Feng Wang and Xiao‐Fei Gao analysed data. WSY performed the bioinformatics analysis. Feng Wang and Shu‐Yu Wang wrote the manuscript. Shao‐Liang Chen and Xiao‐Fei Gao revised the manuscript. Xiao‐Fei Gao, Jun‐Jie Zhang, and Shao‐Liang Chen designed and supervised experiments.

## CONFLICT OF INTEREST STATEMENT

The authors declare no conflict of interest.

## ETHICS STATEMENT

Written informed consent was obtained from all patients. The study was approved by the Ethics Committee of Nanjing First Hospital and conformed to the principles outlined in the Declaration of Helsinki. The study was approved by the Institutional Animal Care and Use Committee of Nanjing First Hospital and conformed to the Guide of the Care and Use of Laboratory Animals (US National Institutes of Health).

## Supporting information



Supporting Information

## Data Availability

The raw RNA‐seq data in this study has been uploaded to the SRA database with accession number PRJNA1062019. The publicly available RNA‐seq data and scRNA‐seq data re‐analysed in this study has been deposited in the Gene Expression Omnibus data base or SRA database under the accession codes GSE92506, GSE83476, GSE266437, GSE197366, GSE159677, GSE234077, GSE224273, GSE155512, PRJNA802316, GSE131778, GSE184073, GSE196943, GSE201091, GSE166676, GSE155468, GSE216860, PRJNA722117, PRJNA646233. The corresponding link is available in Table S2. Source data are provided with this paper. Raw data will be available upon request.
