# Supplementary material for: Disturbed shear stress promotes atherosclerosis through TRIM21‐regulated MAPK6 degradation and consequent endothelial inflammation
Source: Clin Transl Med. 2025 Jan 6;15(1):e70168. doi: 10.1002/ctm2.70168 (PMC11705438; doi:10.1002/ctm2.70168)
Supplement: Supplementary file 1 — Supporting Information [file CTM2-15-e70168-s001.docx]

**
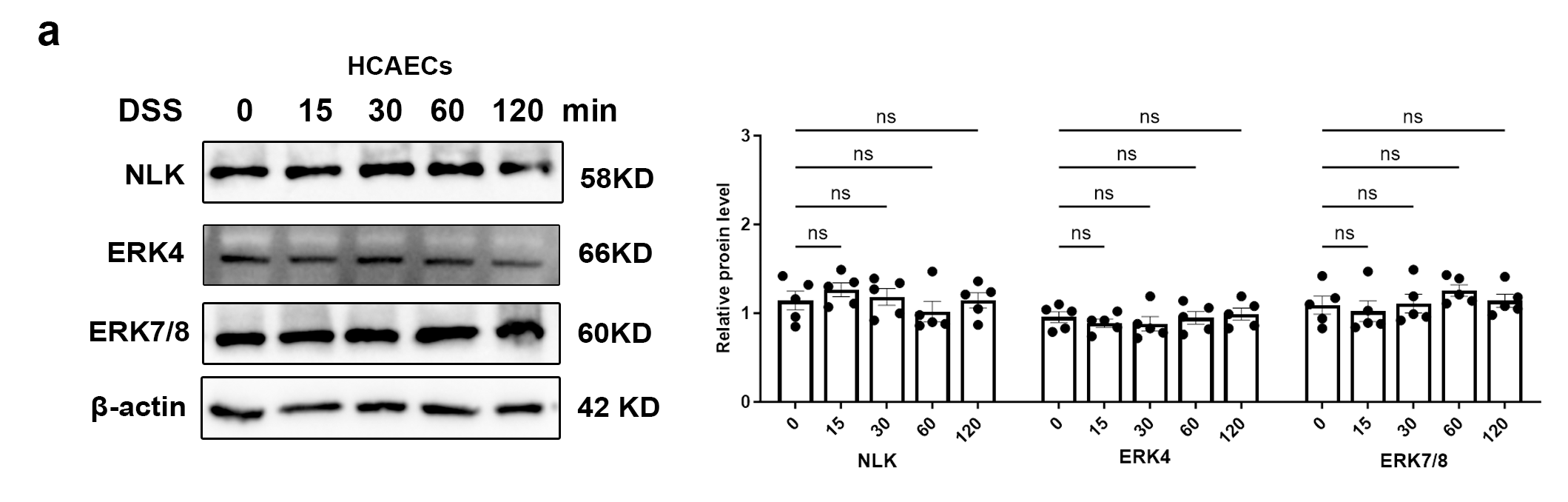
**

**Fig. S1. The levels of other MAPK family members did not change in response to DSS.** HCAECs were stimulated by DSS (0, 15, 30, 60, 120 min), protein levels of NLK, ERK4 and ERK7/8 were detected (n=5). ns=no significance. One-way ANOVA with the Dunnett multiple comparison test was applied.


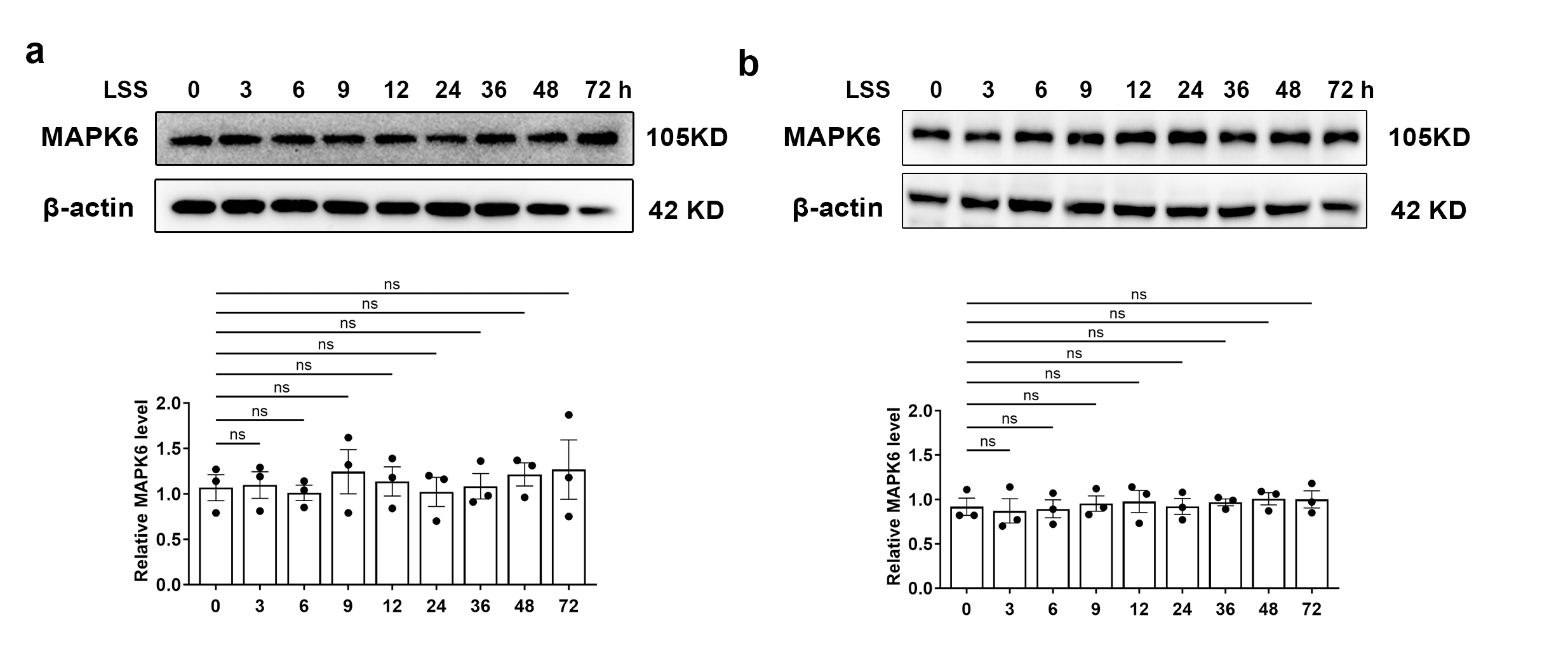


**Fig. S2. The protein of MAPK6 were stable under LSS. a** HUVECs and **b** HCAECs were stimulated by LSS for 0, 3, 6, 9, 12, 24, 36, 48, 72 h, and the MAPK6 protein was detected (n=5). ns=no significance. One-way ANOVA with the Dunnett multiple comparison test was applied.


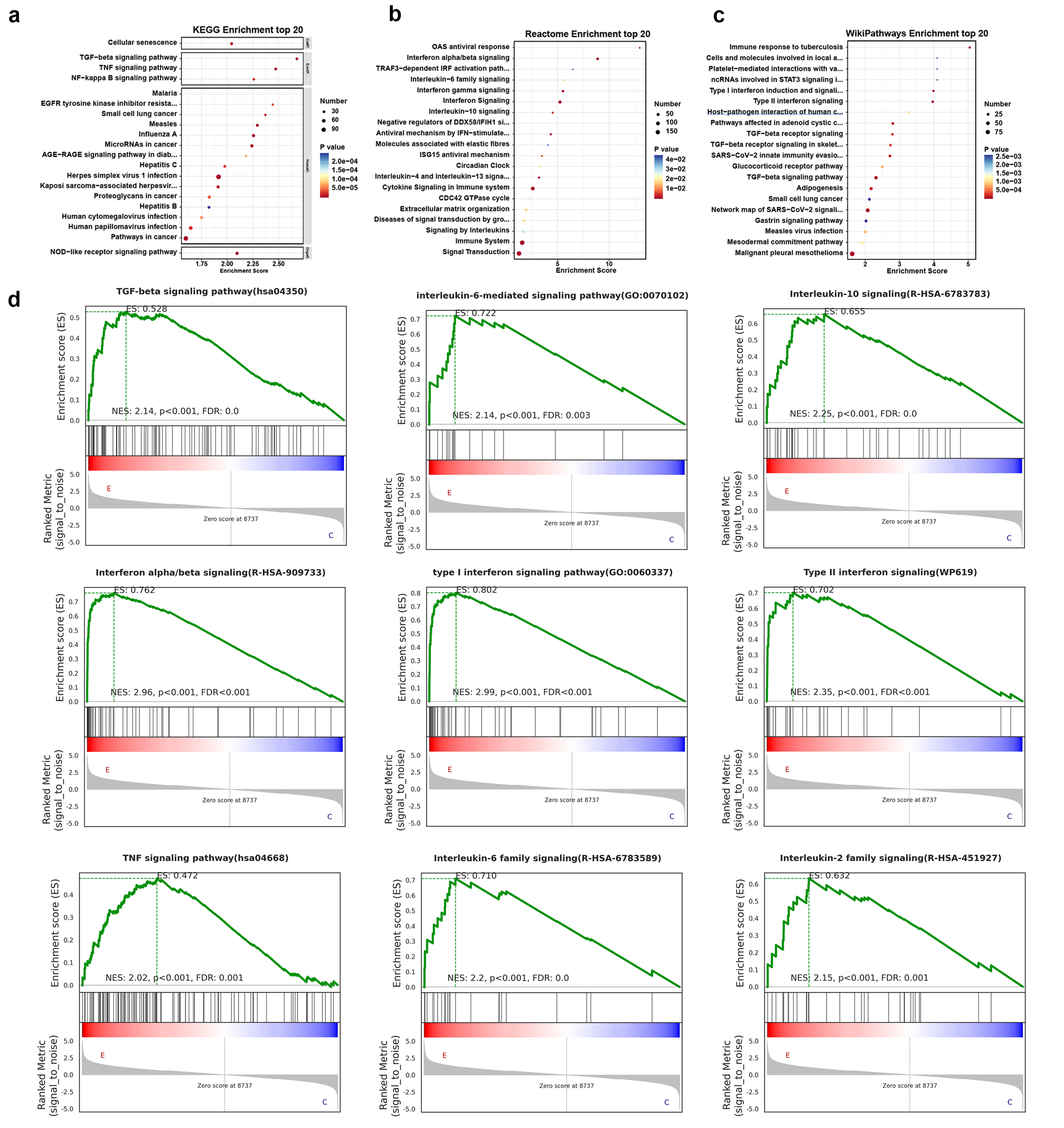


**Fig. S3. Enrichment pathways identified via RNA-seq analysis. a** KEGG, **b** Reactome, and **c** WikiPathways enrichment of the top-20 enrichment pathways of up-regulated genes in HUVECs transfected with MAPK6si compared to the counters. **d** GSEA analysis of genes in HUVECs transfected with MAPK6si and the counters. The threshold adj q-value < 0.05 and |log_2_FC| > 1 were used for identifying DEGs.


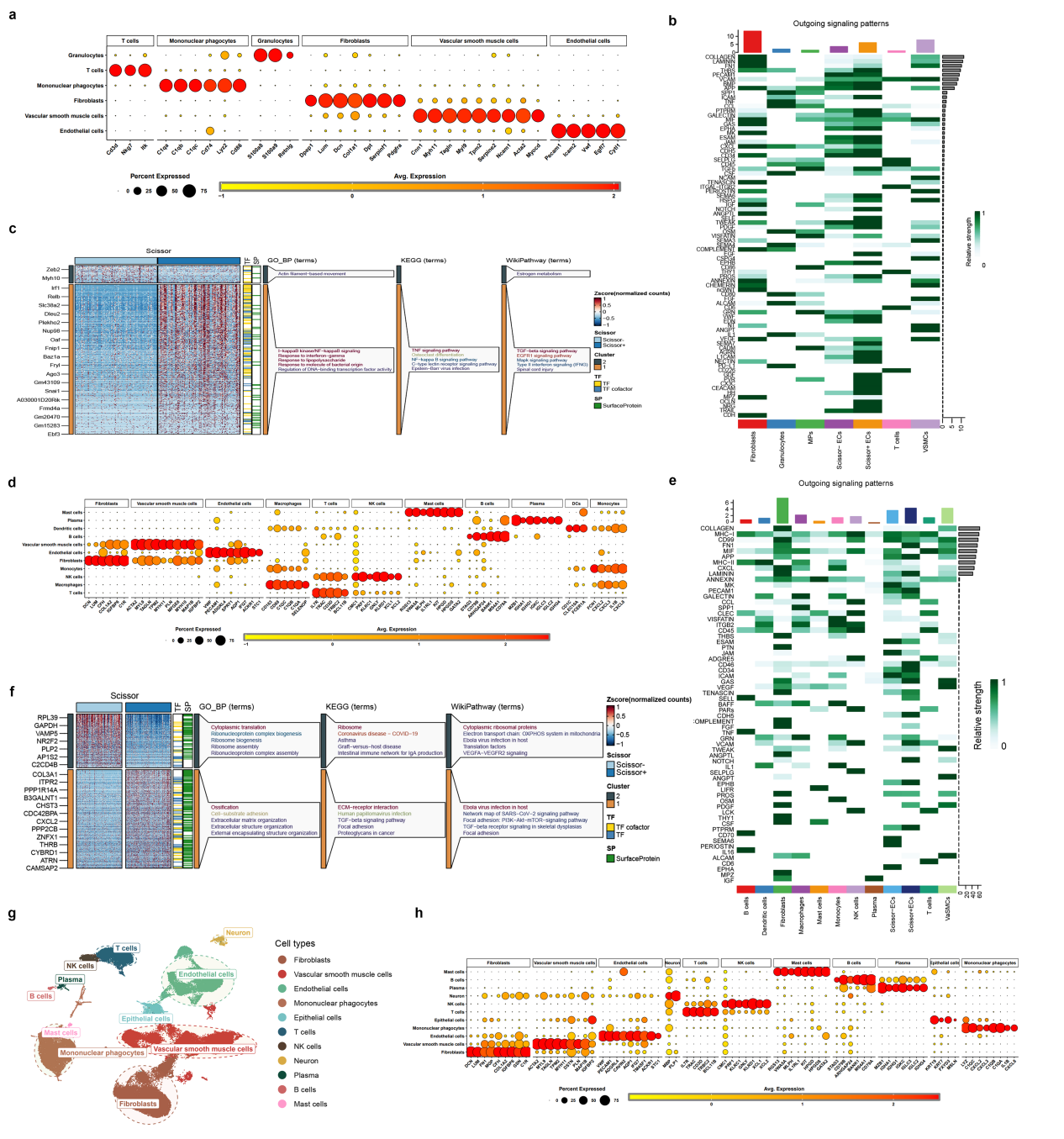


**Fig. S4. In-depth analysis of human normal arteries or plagues and mice PCL model. a** Dot plot of marker genes by major cell compartment in PCL or Control. **b** Outing signalling patterns between different cell types in PCL or Control. **c** Differential analysis and enrichment analysis between Scissor+ ECs and Scissor- ECs in PCL or Control. The threshold q-value < 0.05 and |log_2_FC| > 1 were used for identifying DEGs. **d** Dot plot of marker genes by major cell compartment in plagues. **e** Outing signalling patterns between different cell types in plagues. **f** Differential analysis and enrichment analysis between Scissor+ ECs and Scissor- ECs in plagues. The threshold adj p-value < 0.05 and |log_2_FC| > 1 were used for identifying DEGs. **g** UMAP embeddings of integrated scRNA-seq gene expression data of 12 samples from 4 normal human arteries single-cell datasets after pretreatment (see Methods). **h** Dot plot of marker genes by major cell compartment in arteries.


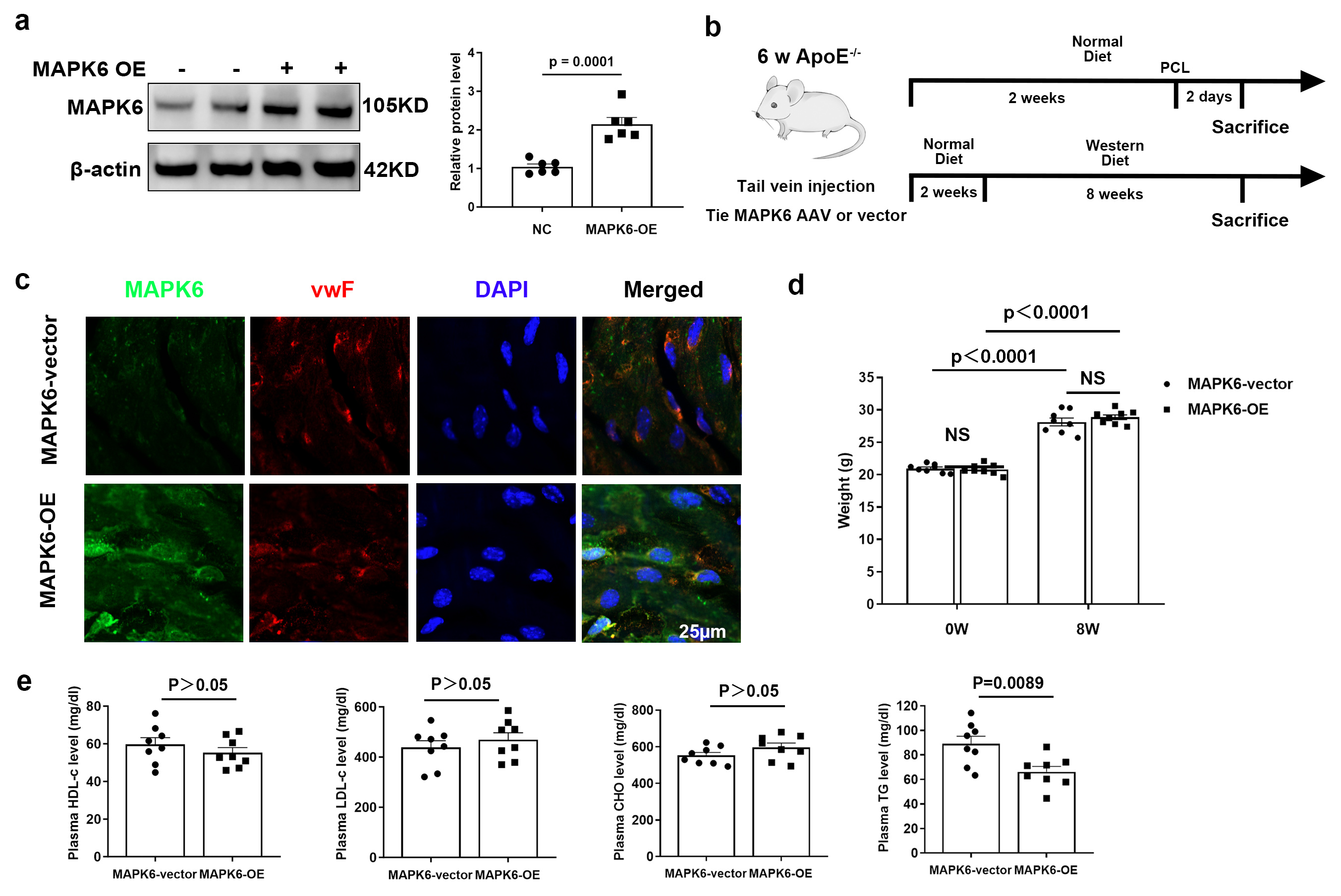


**Fig. S5. Endothelium-specific overexpression of MAPK6. a** MAPK6 plasmid was verified in HUVECs (n=6). **b** Schematic diagram of mouse animal modeling process. **c** Aortic *enface* staining of MAPK6. Scale bar: 25 μm. Green: MAPK6; Red: vWF; Blue: DAPI. **d** Baseline body weight and body weight after 8 weeks of WD feeding (n=8). **e** HDL-c, LDL-c, TC and TG levels after 8 weeks of WD feeding (n=8). ns=no significance. A two-tailed Student’s t test was applied for **a** and **e**. One-way ANOVA with the Tukey multiple comparison test was applied for **d**.


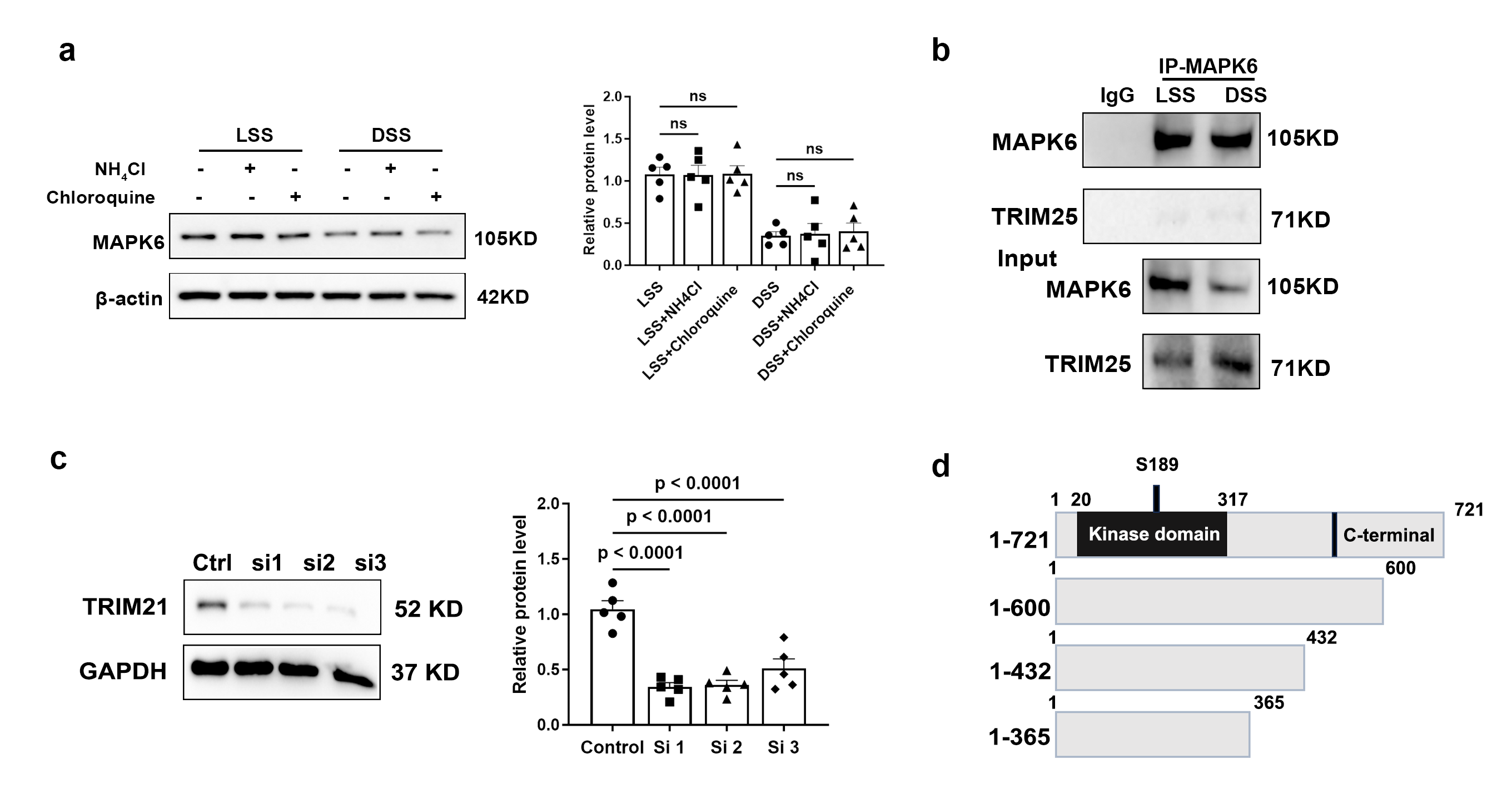


**Fig. S6. Supplement material for ubiquitination degradation. a** MAPK6 protein level of HUVECs under LSS or DSS (6 h), with or without NH_4_Cl, Chloroquine (n=5). **b** MAPK6 and TRIM25 in the input and immunoprecipitates of IgG or anti-MAPK6 antibody in HUVECs treated with LSS or DSS (n=5). **c** Validation of TRIM21 interference efficiency (n=5). **d** Schematic representation of plasmid construction of different MAPK6 subclones. ns=no significance. One-way ANOVA with the Dunnett multiple comparison test was applied.


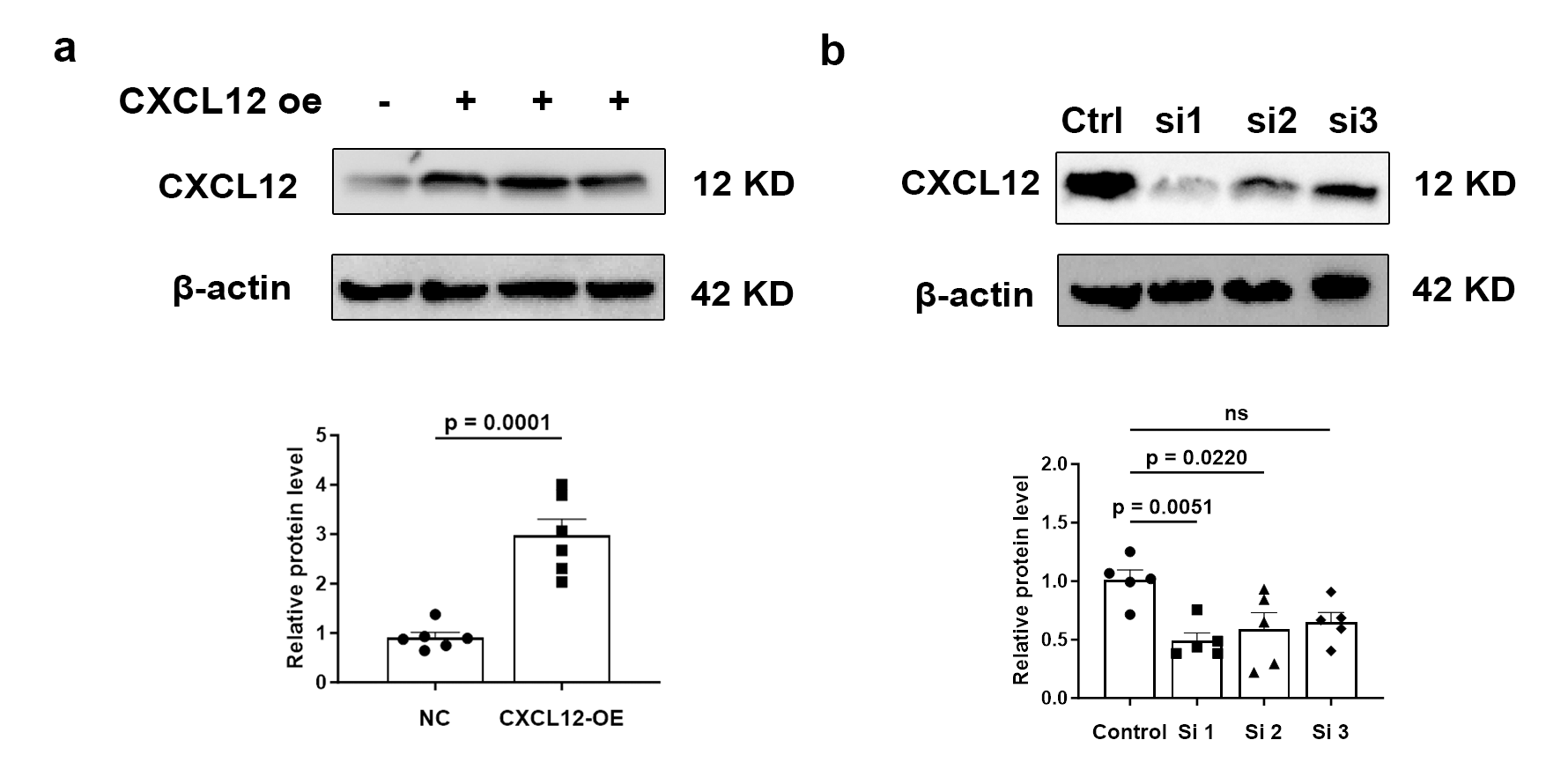


**Fig. S7. Overexpression and interference of CXCL12.** **a** CXCL12 overexpression was verified in HUVECs (n=6). **b** Validation of CXCL12 interference efficiency (n=5). ns=no significance. A two-tailed Student’s t test was applied to **a**. One-way ANOVA with the Dunnett multiple comparison test was applied for **b**.


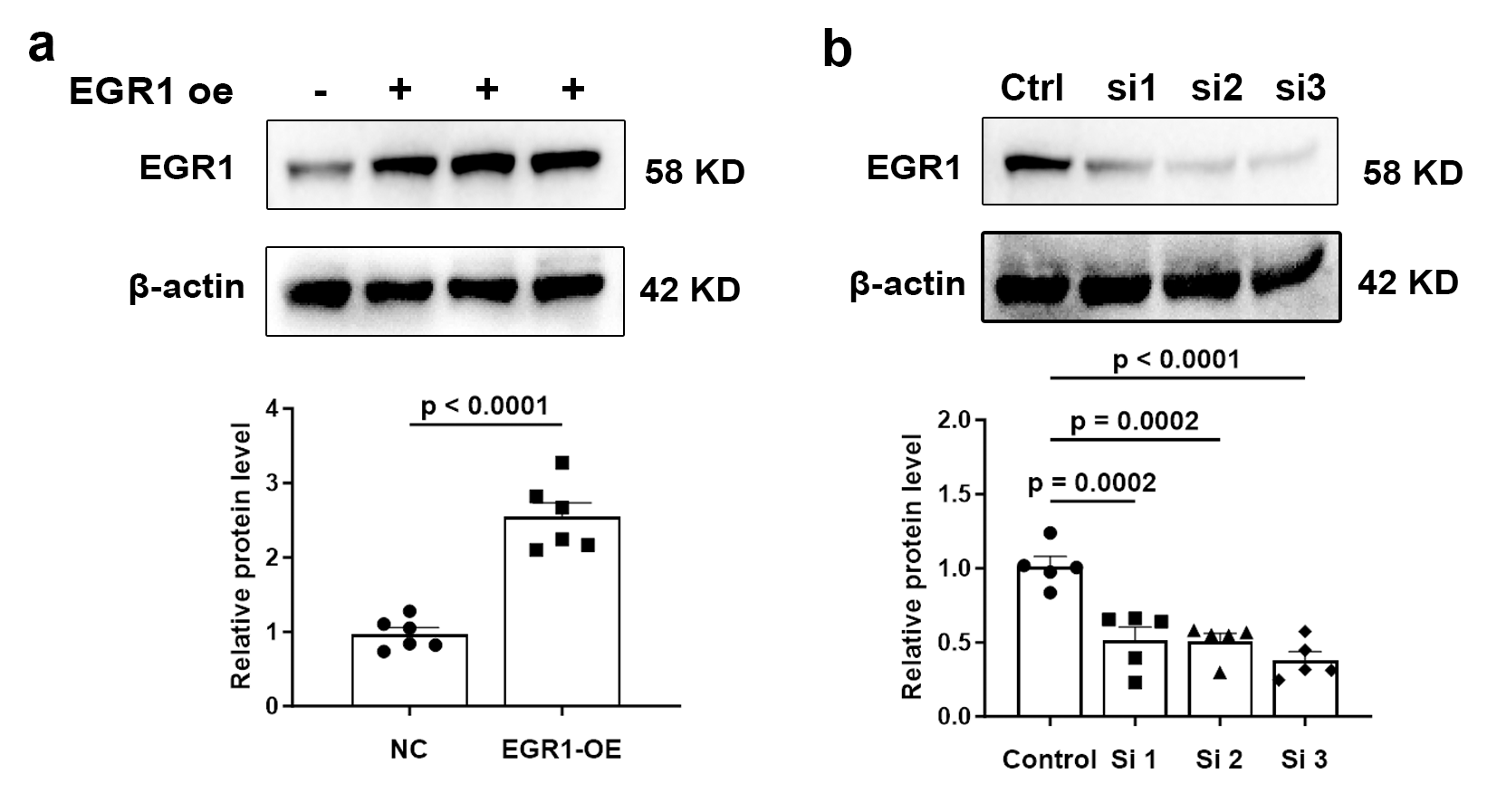


**Fig. S8. Overexpression and interference of EGR1**. **a** EGR1 overexpression was verified in HUVECs (n=6). **b** Validation of EGR1 interference efficiency (n=5). A two-tailed Student’s t test was applied to **a**. One-way ANOVA with the Dunnett multiple comparison test was applied for **b**.


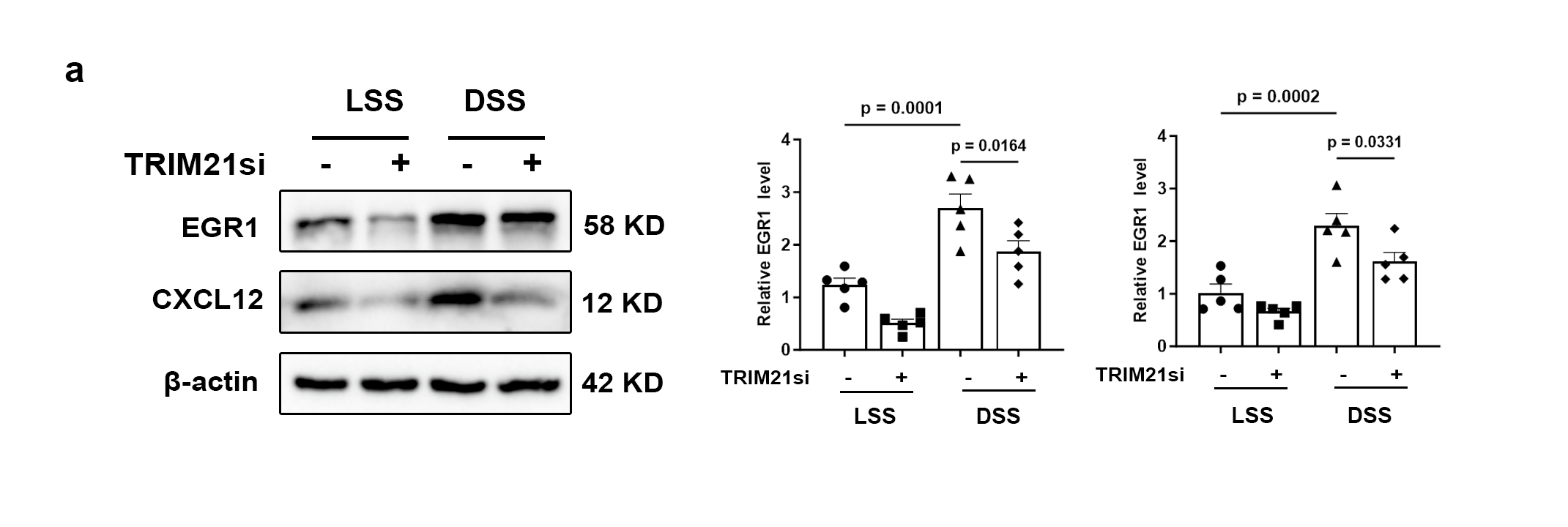


**Fig. S9. The effect of TRIM21 on EGR1-CXCL12 pathway**. Protein levels of CXCL12 and EGR1 in HCAECs which transfected with the siTRIM21 or NC and treated with LSS or DSS (n=5) . One-way ANOVA with the Tukey multiple comparison test was applied.


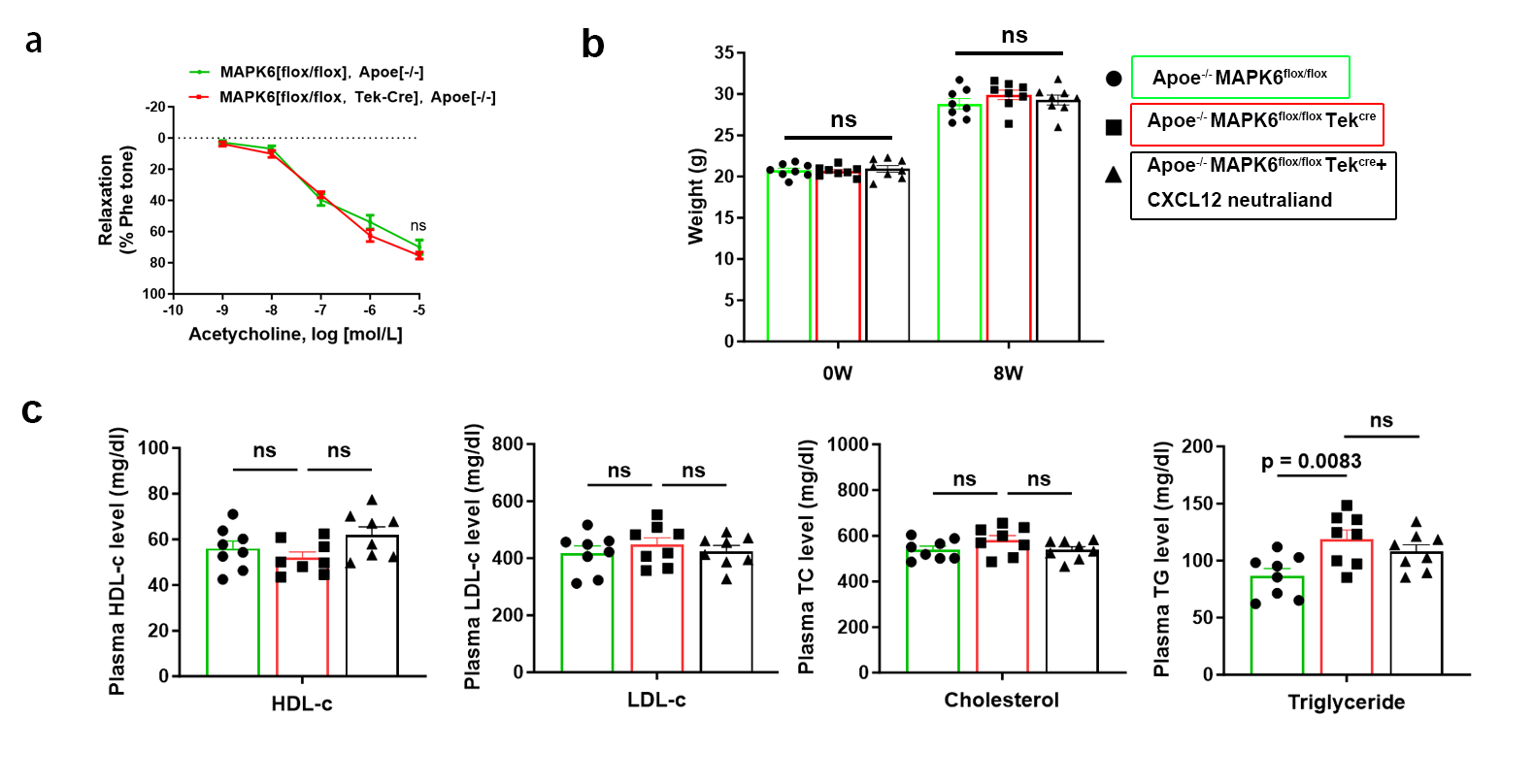


**Fig. S10. Supplement material for conditional knockout of endothelial MAPK6. a** Concentration-response curves of aortic endothelial-dependent relaxation (n=6). **b** HDL-c, LDL-c, TC and TG levels of mice after 8 weeks of WD feeding (n=8). **c** Baseline body weight and body weight after 8 weeks of WD feeding (n=8). ns=no significance. One-way ANOVA with the Tukey multiple comparison test was applied.

**Tables S1. Differential analysis results of MAPK family in GSE197366**

| Gene | p-value | LSMean d-flow | LSMean s-flow | Fold change |
| --- | --- | --- | --- | --- |
| Mapk1 | 0.424 | 0.929 | 0.910 | -1.099 |
| Mapk3 | 0.989 | 1.000 | 0.967 | -1.034 |
| Mapk6 | 0.087 | 0.568 | 2.390 | 2.390 |
| Mapk7 | 0.252 | 0.801 | 0.423 | -2.364 |
| Mapk8 | 0.241 | 0.790 | 0.796 | -1.256 |
| Mapk9 | 0.893 | 1.000 | 0.905 | -1.104 |
| Mapk10 | 0.650 | 1.000 | 1.751 | 1.751 |
| Mapk11 | 0.894 | 1.000 | 1.387 | 1.387 |
| Mapk12 | 0.080 | 20.576 | 27.692 | -1.346 |
| Mapk13 | 0.244 | 3.555 | 5.336 | -1.501 |
| Mapk14 | 0.093 | 4.550 | 7.058 | -1.551 |
| Nlk | 0.248 | 64.006 | 73.167 | -1.143 |

**Tables S2. Details and quality controls of RNA-seq and scRNA-seq datasets**

| Data | Species | Sample | State | Number | Quality control | Link |
| --- | --- | --- | --- | --- | --- | --- |
| GSE159677 | Human | Carotid arteries | Atherosclerotic | 6 | genes that were expressed in less than 3 cells were excluded, remove cells with >10% mitochondrial mRNA , <200 or >4000 nCount_RNA | https://www.ncbi.nlm.nih.gov/geo/query/acc.cgi?acc=GSE159677 |
| GSE234077 | Human | Carotid arteries | Atherosclerotic | 3 | genes that were expressed in less than 3 cells were excluded, remove cells with >10% mitochondrial mRNA, <200 or > 10000 nCount_RNA , <200 or >10,000 nFeature_RNA | https://www.ncbi.nlm.nih.gov/geo/query/acc.cgi?acc=GSE234077 |
| GSE224273 | Human | Carotid arteries | Atherosclerotic | 6 | genes that were expressed in less than 3 cells were excluded, remove cells with >10% mitochondrial mRNA, <200 or > 3000 nCount_RNA | https://www.ncbi.nlm.nih.gov/geo/query/acc.cgi?acc=GSE224273 |
| GSE155512 | Human | Carotid arteries | Atherosclerotic | 3 | genes that were expressed in less than 10 cells were excluded, remove cells with >10% mitochondrial mRNA, <200 or > 4000 nCount_RNA , >20,000 nFeature_RNA | https://www.ncbi.nlm.nih.gov/geo/query/acc.cgi?acc=GSE155512 |
| PRJNA802316 | Human | Carotid arteries | Atherosclerotic | 2 | genes that were expressed in less than 10 cells were excluded, remove cells with >25% mitochondrial mRNA, <200 or > 5000 nCount_RNA , >30,000 nFeature_RNA | https://www.ncbi.nlm.nih.gov/bioproject/PRJNA802316 |
| GSE131778 | Human | Coronary arteries | Atherosclerotic | 8 | genes that were expressed in less than 5 cells were excluded, remove cells with >7.5% mitochondrial mRNA, <500 or > 3500 nCount_RNA | https://www.ncbi.nlm.nih.gov/geo/query/acc.cgi?acc=GSE131778 |
| GSE184073 | Human | Coronary arteries | Atherosclerotic | 2 | genes that were expressed in less than 3 cells were excluded, remove cells with >15% mitochondrial mRNA, <200 or > 5000 nCount_RNA | https://www.ncbi.nlm.nih.gov/geo/query/acc.cgi?acc=GSE184073 |
| GSE196943 | Human | Coronary arteries | Atherosclerotic | 12 | genes that were expressed in less than 3 cells were excluded, genes that were expressed in less than 5 cells were excluded, remove cells with >15% mitochondrial mRNA, <300 or > 2500 nCount_RNA | https://www.ncbi.nlm.nih.gov/geo/query/acc.cgi?acc=GSE196943 |
| GSE201091 | Human | Aortas | Normal | 1 | genes that were expressed in less than 3 cells were excluded, remove cells with >10% mitochondrial mRNA, <200 or > 6000 nCount_RNA | https://www.ncbi.nlm.nih.gov/geo/query/acc.cgi?acc=GSE201091 |
| GSE166676 | Human | Aortas | Normal | 2 | genes that were expressed in less than 3 cells were excluded, remove cells with >25% mitochondrial mRNA, <200 or > 2500 nCount_RNA | https://www.ncbi.nlm.nih.gov/geo/query/acc.cgi?acc=GSE166676 |
| GSE155468 | Human | Aortas | Normal | 3 | genes that were expressed in less than 3 cells were excluded, remove cells with >10% mitochondrial mRNA, <200 nCount_RNA | https://www.ncbi.nlm.nih.gov/geo/query/acc.cgi?acc=GSE155468 |
| GSE216860 | Human | Aortas | Normal | 6 | genes that were expressed in less than 3 cells were excluded, remove cells with >25% mitochondrial mRNA, <200 or > 6000 nCount_RNA | https://www.ncbi.nlm.nih.gov/geo/query/acc.cgi?acc=GSE216860 |
| PRJNA722117 | Mouse | Carotid arteries | D-flow or s-flow | 1 vs 1 | genes that were expressed in less than 3 cells were excluded, remove cells with >10% mitochondrial mRNA, <200 or > 5000 nCount_RNA | https://www.ncbi.nlm.nih.gov/bioproject/PRJNA722117 |
| PRJNA646233 | Mouse | Carotid arteries | D-flow or s-flow | 2 vs 2 | genes that were expressed in less than 3 cells were excluded, remove cells with >10% mitochondrial mRNA, <200 or > 7600 nCount_RNA | https://www.ncbi.nlm.nih.gov/bioproject/?term=PRJNA646233 |
| GSE92506 | Human | HUVECs | OS or PS | 8 vs 8 | - | https://www.ncbi.nlm.nih.gov/geo/query/acc.cgi?acc=GSE92506 |
| GSE83476 | Human | HCAECs | OS or LS | 4 vs 4 | - | https://www.ncbi.nlm.nih.gov/geo/query/acc.cgi?acc=GSE83476 |
| GSE266437 | Human | HAECs | DF or UF | 3 vs 3 | - | https://www.ncbi.nlm.nih.gov/geo/query/acc.cgi?acc=GSE266437 |
| GSE197366 | Mouse | Carotid arteries (endothelial-enriched) | D-flow or s-flow | 3 vs 3 | - | https://www.ncbi.nlm.nih.gov/geo/query/acc.cgi?acc=GSE197366 |

**Tables S3. Sequences of siRNA**

| Genes | Sequences | Species |
| --- | --- | --- |
| MAPK6-1 | 5’- GCGUAAUUCCAGUUUACAUTT -3’  5’-AUGUAAACUGGAAUUACGCTT -3’ | Human |
| MAPK6-2 | 5’- CAGGUGCACAUGAACUUGAACAGAU-3’  5’- AUCUGUUCAAGUUCAUGUGCACCUG-3’ | Human |
| MAPK6-3 | 5’-CAGAGCAUGAUUGGCCUGUACAUAA-3’  5’-UUAUGUACAGGCCAAUCAUGCUCUG-3’ | Human |
| TRIM21-1 | 5’- GCAGAGCAUACCUGGAAAUTT -3’  5’-AUUUCCAGGUAUGCUCUGCTT -3’ | Human |
| TRIM21-2 | 5’-GAGAAAGAUGGGAAGGCCCUUUGCU-3’  5’-AGCAAAGGGCCUUCCCAUCUUUCUC-3’ | Human |
| TRIM21-3 | 5’-AGCAAAGGGCCUUCCCAUCUUUCUC-3’  5’-UUUCCAGGACAAUUAUCACCUCCUG-3’ | Human |
| CXCL12-1 | 5’-CACGUAGCAGCUUAGACUA-3’  5’-UAGUCUAAGCUGCUACGUG-3’ | Human |
| CXCL12-2 | 5’-AUGGCUUUCGAAGAAUCGGCAUGGG-3’  5’-CCCAUGCCGAUUCUUCGAAAGCCAU-3’ | Human |
| CXCL12-3 | 5’-ACUCAUUGGUUCCUUUAAGGG-3’  5’-CUUAAAGGAACCAAUGAGUCC-3’ | Human |
| EGR1-1 | 5’-AGAGGCAUACCAAGAUCCA-3’  5’-UGGAUCUUGGUAUGCCUCU-3’ | Human |
| EGR1-2 | 5’-GTGACTGTTTGGCTTATAATT-3’  5’-TTATAAGCCAAACAGTCACTT-3’ | Human |
| EGR1-3 | 5’-CAACGACAGCAGUCCCAUUtt-3’  5’-AAUGGGACUGCUGUCGUUGga-3’ | Human |
| Control | 5’-UUCUCCGAACGUGUCACGU-3’  5’-ACGUGACACGUUCGGAGAA-3’ | Human |

**Tables S4. Antibodies used for western blotting and IF**

| Target | Catalog no. | Host specie | Company | Dilution |
| --- | --- | --- | --- | --- |
| MAPK6 | SC-365234 | Mouse | Santa Cruz Biotechnology | 1:500 (1:100 for IF) |
| MAPK6 | SC-374239 | Mouse | Santa Cruz Biotechnology | 1:500 (1:100 for IF,1:250 for IP) |
| MAPK6 | Ab53277 | Rabbit | Abcam | 1:1000 |
| TRIM21 | SC-25351 | Mouse | Santa Cruz Biotechnology | 1:500 |
| VWF | SC-271409 | Mouse | Santa Cruz Biotechnology | 1:100 for IF |
| ERK7/8 | 13452-1-AP | Rabbit | Proteintech | 1:1000 |
| ERK4 | NBP1-58082 | Rabbit | Novus Biologicals | 1:1000 |
| NLK | SC-271323 | Mouse | Santa Cruz Biotechnology | 1:1000 |
| Myc-Tag | 2276 | Rabbit | Cell Signaling Technology | 1:1000 (1:250 for IP) |
| His-Tag | 12698 | Rabbit | Cell Signaling Technology | 1:1000 (1:50 for IP) |
| GAPDH | 5174 | Rabbit | Cell Signaling Technology | 1:1000 |
| β-actin | 4970 | Rabbit | Cell Signaling Technology | 1:1000 |
| p-MAPK6 | Ab74032 | Rabbit | Abcam | 1:1000 |
| Ub | Ab134953 | Rabbit | Abcam | 1:1000 |
| Ub K48 | Ab140601 | Rabbit | Abcam | 1:1000 |
| Ub K63 | Ab179434 | Rabbit | Abcam | 1:1000 |
| ICAM1 | Ab109361 | Rabbit | Abcam | 1:1000 (1:200 for IF) |
| VCAM1 | 11444-1-AP | Rabbit | Proteintech | 1:1000 |
| NF-kb p65 | 8242 | Rabbit | Cell Signaling Technology | 1:1000 (1:100 for CHIP) |
| TRIM21 | SC-48430 | Mouse | Santa Cruz Biotechnology | 1:500 (1:250 for IP) |
| mcherry | Ab213511 | Rabbit | Abcam | 1:1000 (1:20 for IP) |
| H3 | Ab1791 | Rabbit | Abcam | 1:1000 |
| EGR1 | Ab6054 | Rabbit | Abcam | 1:1000 |

**Tables S5. Primers used for PCR**

| Gene name | Primer sequences | Species | NCBI accession numbers |
| --- | --- | --- | --- |
| ICAM1 | F: GTGATGCTCAGGTATCCATCCA  R: CACAGTTCTCAAAGCACAGCG | Mouse | NM_010493.3 |
| ICAM1 | F: CAGTGACCATCTACAGCTTTCCGG  R: GCTGCTACCACAGTGATGACAA | Human | NM_000201.3 |
| VCAM1 | F: TTGGGAGCCTCAACGGTACT  R: GCAATCGTTTTGTATTCAGGGGA | Mouse | NM_011693.3 |
| VCAM1 | F: GATACAACCGTCTTGGTCAGCCC  R: CGCATCCTTCAACTGGGCCTT | Human | NM_001199834.2 |
| MAPK6 | F: TGTCAAACATGCTCTACGTGAAA  R: TCGTCTGTTAATTGGCTTCCAC | Human | NM_002748 |
| 18S | F: GGCCGTTCTTAGTTGGTGGAGCG  R: CTGAACGCCACTTGTCCCTC | Human/  Mouse | - |
| CXCL12(ChIP assays) | F: CTCATACCAGGGAGCCATAT  R: TCCTCTAACCCAAATCTCTCC | Human | NC_000010.11 |
